# Preoperative classification of histopathological growth patterns in colorectal liver metastases using prospective 18 F-FDG PET/CT

**DOI:** 10.1007/s10585-026-10441-x

**Published:** 2026-11-07

**Authors:** J. Jonsson, A. Ling, J. Axelsson, M. Lundin, P. Vermeulen, K. Riklund, H. Nyström

**Affiliations:** 1 Department of Diagnostics and Intervention, Surgery, Umeå University, Umeå, Sweden; 2 Department of Medical Biosciences, Pathology, Umeå University, Umeå, Sweden; 3 Department of Diagnostics and Intervention, Radiation Physics, Umeå University, Umeå, Sweden; 4 Department of Statistics, Umeå School of Business, Economics and Statistics (USBE), Statistics Umeå University, Umeå, Sweden; 5 Translational Cancer Research Unit, Oncology Center Antwerp, Ziekenhuis aan de Stroom, Wilrijk, Antwerp, Belgium; 6 Department of Diagnostics and Intervention, Diagnostic Radiology, Umeå University, Umeå, Sweden; 7 Wallenberg Centre for Molecular Medicine, Umeå University, Umeå, Sweden

**Keywords:** Colorectal liver metastases, Histopathological growth pattern, FDG-PET/CT, Texture analysis, Metabolic tumour volume

## Abstract

Colorectal liver metastases (CLM) exhibit distinct histopathological growth patterns (HGPs) at the metastasis-liver interface, clinically relevant for prognosis and potential predictive value. This study investigates the feasibility of using Fluorine-18 Fluorodeoxyglucose Positron Emission Tomography Computed Tomography (^18^F-FDG PET/CT) to preoperatively classify HGPs in CLM and secondly investigate PET parameters as prognostic markers for overall survival (OS). In a prospective study at Umeå University Hospital (June 2013-December 2017), 78 patients with resectable CLM underwent contrast-enhanced liver MRI and ^18^F-FDG PET/CT imaging. HGP classification was performed on tissue sections according to 2017 international guidelines. PET features (SUV_mean_ [standardised uptake value], SUV_max_, metabolic tumour volume [MTV] and first-order texture metrics) were analysed to distinguish encapsulating (eHGP) from replacement (rHGP) patterns. In pretreated patients, multivariate logistic regression including both metabolic and texture parameters identified significant associations for parameters from the fixed level threshold algorithm (VuePoint Sharp IR) with small odds ratios. Overall, PET parameters showed limited ability to discriminate between eHGP and rHGP. OS was superior with eHGP versus rHGP (log-rank *p* = 0.045). In chemonaïve patients, SUV-based metrics showed unadjusted exploratory associations with OS after liver surgery, varying by reconstruction/delineation and were not significant after correction for multiple testing. FDG-PET-derived parameters were not reliably associated with HGP and did not support non-invasive HGP prediction in this cohort. eHGP was associated with superior survival, underscoring the value of HGP assessment for prognostication while alternative non-invasive biomarkers remain to be identified.

**Supplementary Information:**

The online version contains supplementary material available at https://doi.org/10.1007/s10585-026-10441-x.

## Introduction

Colorectal cancer (CRC) remains one of the most common malignancies worldwide, and approximately one-third of patients are diagnosed with colorectal liver metastases (CLM) [1]. Liver surgery is paramount in achieving possibility of lasting cure for CLM patients and the 5-year overall survival exceeds 50% for resected CLM patients [2]. At the metastasis-liver interface, CLM display histopathological growth patterns (HGP) that are clinically relevant [3]. The encapsulating (eHGP) and replacement (rHGP) pattern demonstrate both prognostic [3, 4] and potential predictive value [5]. Furthermore, it has been shown that the rHGP is associated with a higher number of R1 resections [6]. Currently, HGP classification relies on histopathological assessment of resected surgical specimens. The lack of feasible preoperative classification consequently limits the clinical integration of HGP into personalised treatment planning.

The eHGP is marked by a connective tissue rim that distinctly separates the metastasis from surrounding normal parenchyma. Conversely, rHGP have tumour cells infiltrating the liver by replacing the hepatocytes and accessing sinusoids without inducing neovascularisation [5]. Research proposes that eHGP arises when a metastasis fails to acquire rHGP traits, triggering a perimetastatic stromal response resembling benign liver fibrosis [7, 8]. This rim is enriched with immune cells [9], fibroblasts and endothelial cells [5, 8, 10]. Less common patterns include the pushing HGP, in which the metastasis expands by compressing the liver tissue without triggering a desmoplastic reaction, and the sinusoidal HGP, in which cancer cells proliferate within the liver sinusoid lumen [11]. Since these patterns account for less than 10% of HGPs found in patients [11], they are not the focus of our study.

Imaging-based approaches for preoperative HGP classification have the potential to advance clinical research and trial design. As a stratification biomarker, preoperative HGP identification may improve allocation in trials evaluating oncologic therapies and support risk-tailored surgical strategies, such as identifying rHGP patients who may benefit from wider margins or intensified postoperative surveillance. Conventional clinicopathologic and molecular markers provide systemic prognostic insight, but do not characterize the local metastasis-liver interface. Functional imaging such as FDG-PET/CT can complement computed tomography (CT) and magnetic resonance imaging (MRI) by capturing metabolic heterogeneity and biological activity at the metastasis-liver interface, offering additional information relevant to HGP assessment.

Retrospective studies have assessed the feasibility of this classification using contrast-enhanced CT [12, 13] and liver MRI [14–16], as reviewed by Latacz et al. and Li et al. [17, 18]. Cheng et al. applied machine learning to contrast-enhanced CT and achieved high accuracy in distinguishing eHGP from rHGP, reporting AUCs of 0.929 in the training cohort and 0.939 in the external validation cohorts [12]. Han et al. developed an MRI-based radiomics model incorporating tumour and metastasis-liver interface zones; their combined model with clinical, qualitative imaging and radiomics features achieved an AUC of 0.971 in the training cohort and 0.905 in the external validation cohort highlighting the potential of MRI for HGP prediction [14]. Similarly, Ozaki et al. found that arterial phase peritumoural contrast-to-noise predicted rHGP (AUC 0.920) and was correlated with poorer prognosis [16]. While the prognostic value was evaluated across two cohorts, the diagnostic HGP model was restricted to a single training cohort without further validation. Collectively, these findings highlight the promise of imaging-based HGP assessment across modalities, while heterogeneity in retrospective study designs and HGP cut-off thresholds underscores the need for well-designed prospective investigations.

FDG-PET/CT enables molecular tissue characterisation by quantifying glucose metabolism, which is physiologically high in the liver, heart, and brain and upregulated in highly proliferative tumours. In CLM, decline in FDG-PET/CT metrics have been linked to tumour regression and clinical outcomes: relative changes in SUV_max_ (standardised uptake value) correlate with histologic tumour regression in resected CLM [19], while metabolic tumour volume (MTV) and total lesion glycolysis (TLG) serve as prognostic indicators following medical and surgical interventions [20]. Bohlok et al. reported higher baseline glucose uptake in non-eHGP patients compared with 100% eHGP CLM (mean SUV_max_/SUV_mean(liver)_ 4.5 ± 2 for non-eHGP and 3.4 ± 1.7 for eHGP [*p* = 0.009]) [21]. In the study by Bohlok et al. 80% of patients received preoperative chemotherapy after PET/CT, which may cause HGP conversion between PET/CT and liver surgery and additionally make results hard to translate to treatment-naïve CLM patients. To our knowledge, the study by Bohlok et al. remains the only PET/CT-focused study published in HGP research, and no prospective studies have yet been conducted in this field.

The overarching aim of this prospective exploratory study was to investigate if FDG-PET/CT can be used in the preoperative setting to non-invasively identify the two most common HGP in CLM patients. Our primary aim was to identify FDG-PET/CT imaging features distinguishing eHGP from rHGP in chemonaïve and pretreated cohorts. Our secondary aim was to investigate if PET parameters (SUV_max_, SUV_mean_ or MTV) correlate with overall survival following liver surgery.

## Material and method

### Study design and patient cohort

Umeå university hospital is a tertiary hepato-pancreatico-biliary centre serving the four northern regions of Sweden. For this study, patients with CLM were prospectively enrolled from the multidisciplinary team conference (MDT) of CLM if deemed eligible between 1st June 2013 to 31st December 2017. Standard staging included contrast-enhanced liver MRI and CT of the thorax and abdomen; all patients additionally underwent ^18^F-FDG PET/CT according to national guidelines. Patients with CLM were included in the study when deemed resectable at MDT. Excluded patients were: (1) patients planned for ablative intervention only, (2) patients who did not give their consent, (3) patients who were not able to conceive written or oral information, and (4) patients evaluated with FDG-PET/CT who later did not undergo liver resection for any reason. Patients who did not undergo liver surgery were excluded from further analysis since no tissue sections could be retrieved for HGP classification.

Throughout the study period, the national guidelines recommended FDG-PET/CT for staging of all patients with metastatic CRC. Clinical data were collected from medical charts. The date of deaths was obtained from the Swedish population register. Prior to analysing the FDG-PET/CT results, we divided the patient cohort into chemonaïve and pretreated groups, since pre-imaging chemotherapy significantly alters metabolic activity, necessitating subgroup analysis [22].

**Fig. 1:**
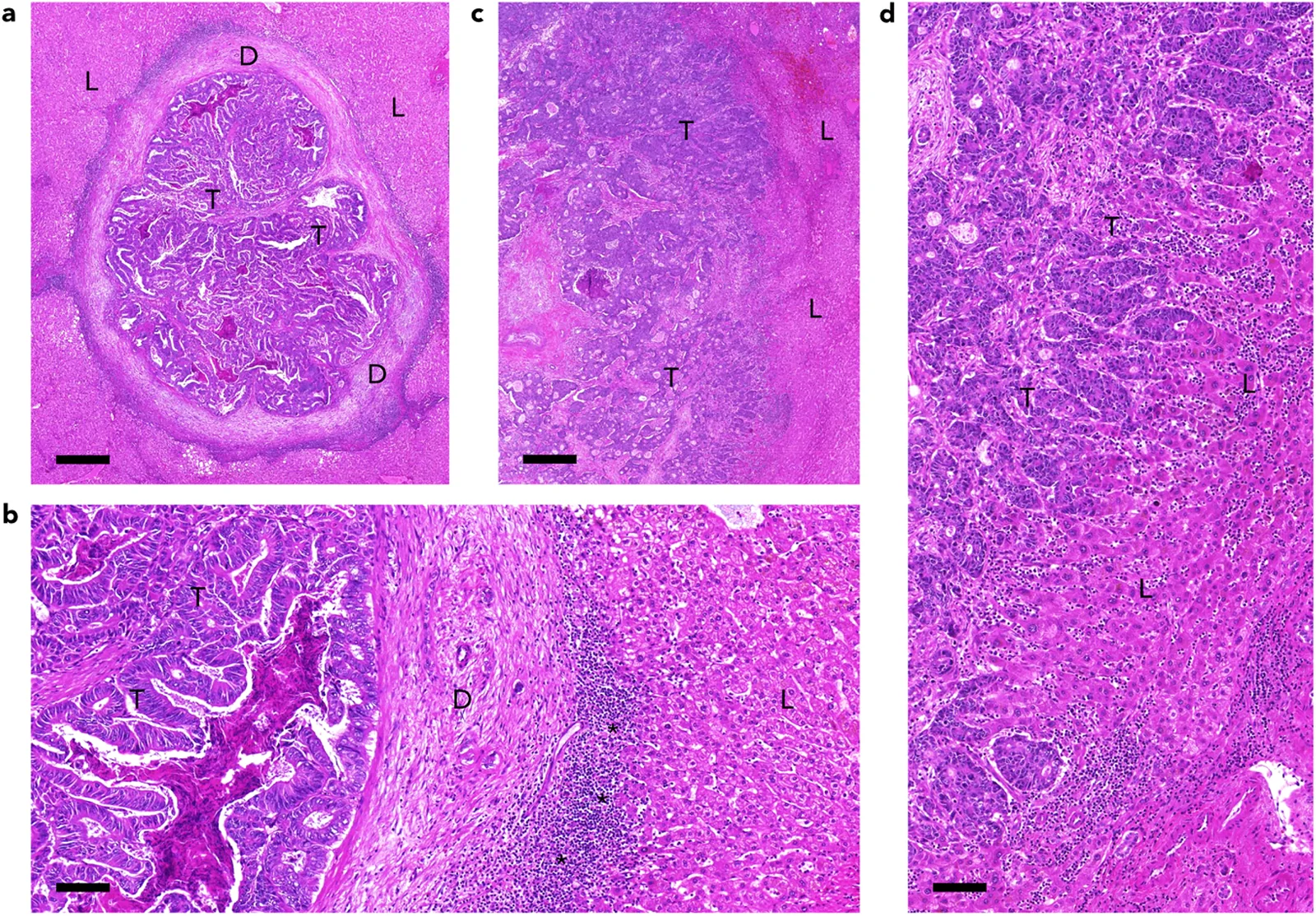
Hematoxylin and eosin images of the histopathological growth pattern (HGP).** a** and **b** encapsulating HGP (eHGP). **a** low magnification (20X) images of the eHGP with a desmoplastic rim separating the tumour cells from the hepatocytes. **b** higher magnification (100X) of the metastasis in a including a dense lymphocyte infiltrate (*).** c** and** d** replacement HGP (rHGP). **c** low magnification (20X) of the rHGP without a demarcated border between the metastasis and the normal liver. **d** high magnification (100X) of the rHGP. D , desmoplastic reaction, L, normal liver parenchyma, T, tumour, *, lymphocyte infiltrate. Scale bar = 500 μm (**a** and **c**), 100 μm (**b** and **d**)

### Classification of histopathological growth patterns in colorectal liver metastases

The HGP classification was performed on haematoxylin & eosin-stained formalin-fixed paraffin-embedded (FFPE) tissue sections from CLM as reported by the 2017 and 2022 International Consensus Guidelines for scoring the HGP of liver metastasis [3, 11]. The primary scoring followed the 2017 guidelines using the predominant (> 50%) cut-off. Figure 1 presents representative tissue sections displaying the eHGP and the rHGP. All available tissue sections involving any metastasis-liver interface were retrieved from the local biobank and included in the HGP classification. The sections were scanned on a Panoramic 250 Flash, 3DHistech (3DHISTECH Ltd., Budapest, Hungary) and digitised. The HGP classification was performed by JJ, AL and PV: a Ph.D student without prior experience of HGP scoring; a resident in pathology without prior experience of HGP scoring; a pathologist specialised in gastrointestinal pathology with > 10 years of experience in scoring the HGPs. All three persons were blinded to clinical data. Multiple consensus meetings were held to resolve any disagreement and to reach agreement in the HGP classification.

### Fluorine-18 fluorodeoxyglucose positron emission tomography protocol

^18^F-FDG PET/CT-data was acquired on a General Electric Discovery 690, WI, US. The patients had been fasting six hours prior to examination. Weight, height, and pre-imaging blood glucose levels were recorded. Whole-body static imaging from the base of the skull to the proximal femur was performed according to clinical routine. A two minute/bed static time-of-flight-mode PET acquisition started 60 min after intravenous injection of ^18^F-FDG at a dose of 4.0 MBq/kg body weight. The CT was acquired with 120 kV, 0.5 s tube rotation time, 2.5 mm slice thickness, 2.5 mm slice width and 30–160 mA automatic tube-current modulation (AutomA). Attenuation-corrected PET images were reconstructed using two algorithms supplied with the scanner:

1. *Vue Point FX*, employing a 6.4 mm filter, 2 iterations, 24 subsets.
2. *Vue Point Sharp IR* resolution recovery algorithm, employing a 4 mm filter, 3 iterations, 24 subsets.

### PET image analysis

The data was exported and anonymised using Dicom2usb (https://www.dicom2usb.com). The image data analysis was performed using the in-house developed image software imlook4d (https://janaxelsson.github.io/imlook4d) version 5.9.1 executed in MATLAB (R2019a, Natick, MA, US, The MathWorks Inc.).

The preoperative MRI and the liver surgeon’s specified liver segment location on the pathologist referral guided the specific segment location of CLM on PET/CT. Metastases were included if there was a visible uptake on PET images with a segmentally concordant MRI position. For each metastasis, a gross volume of interest (VOI) was semi-automated delineated (JJ) on axial view and verified on coronal view. Representative normal liver tissue uptake (SUV_bkg_) was also obtained manually. All VOIs were further validated by an experienced nuclear medicine specialist (KR). The gross VOIs constituted the target volume for subsequent threshold algorithms.

Quantitative functional information from the PET images were calculated in thresholded VOIs of the metastasis. The parameters extracted from the VOIs were SUV_mean_, SUV_max_, MTV, TLG_mean_ and first-order histogram-based texture parameters (standard deviation, skewness, kurtosis, uniformity, entropy). The PET parameters are described in Supplementary Table 1.

**Fig. 2:**
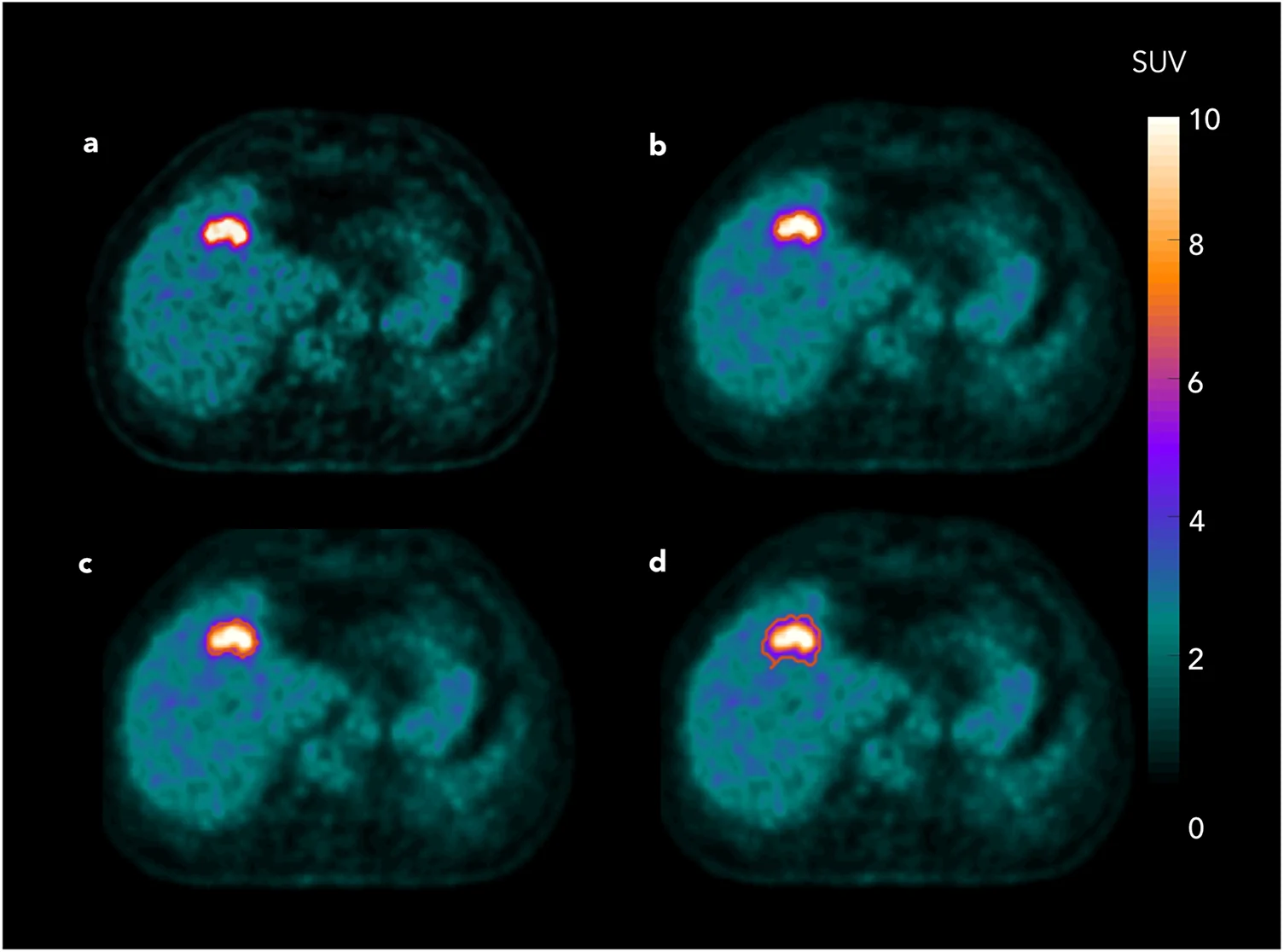
Positron emission tomography images exemplifying the different threshold algorithms for region of interest (ROIs) delineated in red colour.** a** ROI with fixed level threshold algorithm without subtracting the liver background SUV*.** b** ROI with fixed level threshold algorithm with subtracting the liver background SUV.** c** ROI with adaptive threshold algorithm without subtracting the liver background SUV.** d** ROI with adaptive threshold algorithm with subtracting the liver background SUV. *SUV, standardized uptake value. Scalebar representing the SUV values

The VOIs were delineated using both a fixed level threshold and an adaptive threshold algorithm. Both the fixed level and adaptive threshold algorithm were performed in two alternative ways: with and without subtracting the *SUV*_*bkg*_ value from the image. The fixed threshold level was calculated two alternative ways: (1) traditional threshold level = *0.5*⋅*SUV*_*max*_, and (2) threshold 50% above background with the fixed level calculated as *0.5* ⋅ *(SUV*_*max*_ − *SUV*_*bkg*_*) + SUV*_*bkg*_. The adaptive threshold algorithm was performed on normalised SUV images with two alternatives: on a normalised background-subtracted image (*SUV-SUV*_*bkg*_)/*SUV*_*max*_ or on a simple normalised image *SUV*/*SUV*_*max*_. The VOI is calculated from the normalised image with the MATLAB imaging toolbox ‘adaptthresh’ function using a sensitivity of 0.1. The normalisation and adaptive thresholding were performed in one step with the imlook4d built-in menu “SCRIPTS/Adaptive_threshold_within_ROI”. Figure 2 shows the delineation of the region of interests in one patient with the four different threshold algorithms applied.

### Statistical analysis

Statistical analyses were conducted using IBM SPSS Statistics version 25.0 (Mac OS X, Armonk, New York, USA: IBM Corp.) and R version 4.1.1 [23]. Continuous variables were analysed for normal distribution, and differences were calculated using the Mann-Whitney U test. Categorical variables were analysed using the chi-square test of association or Fisher’s exact test when applicable.

Intraclass correlation coefficient (ICC) estimates, and their 95% confidence intervals, were calculated based on mean-rating (k = 3), absolute-agreement, two-way mixed effects model. ICC values were interpreted according to standard guidelines: poor (< 0.5), moderate (0.50–0.75), good (0.75–0.90), and excellent (>0.90) agreement. Interobserver agreement was analysed using intraclass correlation with irr R package version 0.84.1 [24] and dplyr R package version 1.0.10 [25].

Uni- and multivariate mixed-effects logistic regressions evaluated associations between PET parameters and HGP, with the dependent variable (output variable) using the predominant HGP ≥50% as the cut-off. Analyses were performed at the metastasis level with patient included as a random effect to account for clustering of metastases within patients. 95% confidence intervals were used. A* p*-value < 0.10 was considered statistically significant in univariate regression analyses; *p* <0.05 was applied elsewhere. Subgroup analyses were conducted for chemonaïve, and pretreated CLM.* P*-values were adjusted for multiple comparisons using Benjamini-Hochberg false discovery rate procedure, and both unadjusted and adjusted p-values are presented [26]. Different PET reconstruction algorithms (Vue Point FX and Vue Point Sharp IR) and delineation methods were assessed. 

Cox proportional hazards regression models tested associations between PET parameters and overall survival. PET parameters were analysed in chemonaïve and pretreated patients separately, with hazard ratios (HR) and 95% CI reported. Survival probabilities were estimated using the Kaplan-Meier survival curves. Time-to-event was defined as time from liver surgery to death, and patients were censored at the date of last follow-up if the event had not occurred. The log-rank test was used for survival comparisons between HGP groups.

### Ethics

Approval from the Swedish Ethical Review Authority to conduct the study was given (DNR 2009-175 M, amendment 2013-5-23 M and DNR 2019–05690). The participating patients gave their informed and written consent. The study was conducted in accordance with the 1964 Declaration of Helsinki and its later amendments.

**Fig. 3:**
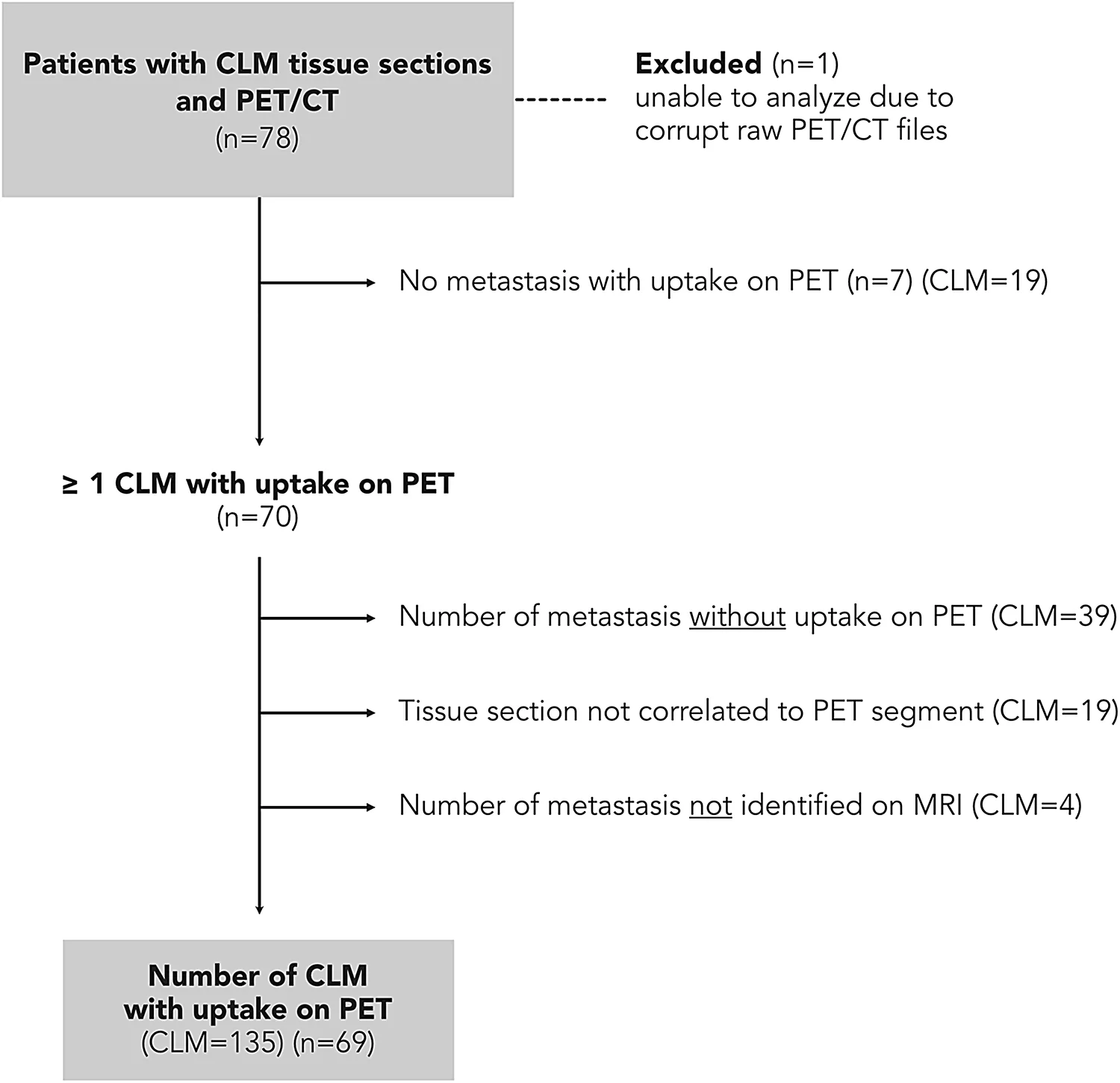
Flowchart of metastasis with both metabolic activity on PET and related tissue section. CLM, colorectal liver metastasis. n, number of patients

## Results

### Clinical characterisation of the cohort

The patient inclusion and exclusion are presented in Supplementary Fig. 1. In our study, 78 patients were evaluated with FDG-PET/CT and underwent resection where the HGP was classified. The characteristics of included patients is presented in Table 1. One PET dataset was corrupt (one patient excluded), leaving 77 patients for final PET-HGP analyses. The PET data analysis was stratified into chemonaïve and pretreated subgroups. Pretreated patients had significantly higher TNM stage at initial diagnosis and a greater CLM burden (Supplementary Table 2).

#### Time between PET/CT and surgery

Chemotherapy was administered prior to FDG-PET/CT imaging in 33 (42%) patients. The median interval between PET/CT and surgery was 15.5 days (range 1–90). Nineteen (24%) patients had an interval exceeding 30 days, and fourteen of these received preoperative chemotherapy. All but three of the patients with intervals > 30 days resided outside Vasterbotten County, indicating that geographic referral patterns may have contributed to delays. One patient was rescheduled for more advanced surgery (hyperthermic intraperitoneal chemotherapy [HIPEC]) after findings of peritoneal carcinomatosis. Another patient received chemotherapy between FDG-PET/CT and surgery, and no metastases were metabolically active in this case.

**Table 1:** Characteristics of included patients

	Patients (*n* = 78)
Age at PET examination/surgery, years (median, [IQR^1^])	66 (58–73)
Gender, Male:Female (n, [%])	51:27 (65%:35%)
Primary tumour	
Localisation, Colon:Rectum (n, [%])	46 (59%):32 (41%)
TNM^2^-stage at CRC^3^ diagnosis	
I (n, [%])	4 (5%)
II (n, [%])	6 (8%)
III (n, [%])	19 (24%)
IV (n, [%])	49 (63%)
*Extrahepatic disease (EHD)*	
Existing extrahepatic disease known at time of surgery (n, [%])	15 (19%)
Previously resected EHD (n, [%])	3 (4%)
Synchronous resection of EHD (n, [%])	19 (13%)
Preoperative CEA^4^-value, µg/L (median, [IQR])	6.15 (2.98-38)
*Colorectal liver metastases*	
Tumour number (median, [IQR])	3 (1–6)
Tumour size in cm (median, [IQR])	3 (2-3.6)*
*Preoperative chemotherapy in relation to liver resection*	
No chemotherapy (n, [%])	45 (57%)
Neoadjuvant (n, [%])	22 (28%)
Downsize/conversion therapy (n, [%])	11 (14%)
Missing information (n, [%])	0 (0%)
*Postoperative chemotherapy in relation to liver resection*	
No adjuvant chemotherapy (n, [%])	36 (46%)
Adjuvant (n, [%])	36 (46%)
Palliative chemotherapy (n, [%])	5 (6%)
Missing information (n, [%])	1(1%)
*Repeated resection*	
No additional resection (n, [%])	57 (73%)
Previous resection (n, [%])	8 (10%)
Repeat resection (n, [%])	13 (17%)

^1^IQR, interquartile range

^2^TNM, tumour node metastasis staging system

^3^CRC, colorectal cancer

^4^CEA, carcinoembryonic antigen

*tumour size, one (*n* = 1) patient presenting with a conglomerate of metastases

### Histopathological growth patterns

A total of 242 CLM from 77 patients were reviewed for HGP classification, of which 27 (11%) CLM were non-classifiable due to absence of viable tumour tissue on tissue sections. Subsequently, 215 CLM were assigned an HGP percentage. In total, 349 tissue sections were assessed, with an average of 4.5 sections per patient (range 1–15). Of the 78 patients included, 76 had at least one classifiable CLM; their characteristics are presented in Table 2. The two patients without classifiable tissue sections had no viable carcinoma (one CLM each) and had not received preoperative chemotherapy.

For this study preformed in 2013–2017, the HGP classification guidelines of 2017 were used [3]. Using the 2022 HGP classification guidelines [11], 100% eHGP was present in 5 (7%) patients and non-eHGP in 71 (93%) patients. Our further analyses were therefore based on the predominant HGP with a > 50% cut-off [3]. Interobserver agreement was high: the ICC was 0.92 (95%CI 0.90–0.94) and 0.88 (0.84–0.91) for the Ph.D student and the resident in pathology compared with the gold standard on initial scoring. After consensus review, ICC improved to 0.97 (95%CI 0.97–0.98) and 0.97 (95%CI 0.96–0.98), respectively.

**Table 2:** Characteristics of patients with classifiable tissue sections using the cut-off ≥ 50% to classify encapsulating and replacement histopathological growth patterns

	eHGP^1^ (*n* = 38)	rHGP^2^ (*n* = 38)	*p*-value
Age at surgery, years (median, [IQR*])	65 (57–73)	66 (58.5–72)	0.763
Gender, Male: Female (n, [%])	25 (66%): 13(34%)	24 (64%): 14 (37%)	0.811
TNM^3^-stage at CRC diagnosis			
I (n, [%])	1 (3%)	2 (5%)	0.943
II (n, [%])	3 (8%)	3 (8%)	
III (n, [%])	10 (26%)	9 (24%)	
IV (n, [%])	24 (63%)	24 (63%)	
*Extrahepatic disease (EHD)*			
Existing EHD known at time of surgery (n, [%])	8 (21%)	7 (18%)	0.773
Previous resected EHD (n, [%])	1 (3%)	2 (5%)	1.000
Synchronous resection of EHD (n, [%])	5 (13%)	5 (13%)	1.000
CEA^4^-value			
Preoperative CEA-value, µg/L (median, [IQR])	4.5 (2.3–14.1)	15.5 (3.3–50)	0.031
*Colorectal liver metastases*			
Tumour number (median, [IQR])	3 (1–7)	3 (1–6)	0.012
Tumour size in cm (median, [IQR])	2.5 (1.7-3.0)	3.0 (2.1-5.0)	0.682
*Preoperative chemotherapy in relation to liver resection*			
No chemotherapy (n, [%])	20 (53%)	22 (58%)	0.561
Neoadjuvant (n, [%])	13 (34%)	9 (24%)	
Downsize/conversion therapy (n, [%])	5 (13%)	7 (18%)	
*Postoperative chemotherapy in relation to liver resection*			
No adjuvant chemotherapy (n, [%])	17 (45%)	17 (45%)	1.000
Adjuvant (n, [%])	18 (47%)	18 (47%)	
Palliative chemotherapy (n, [%])	2 (5%)	3 (8%)	
Missing information (n, [%])	1 (1%)	0 (0%)	
*Repeat resections*			
Repeat resection after current resection (n, [%])	7 (18%)	5 (13%)	0.472
Previous resection (n, [%])	2 (5%)	5 (13%)	

^1^eHGP, encapsulating histopathological growth pattern (HGP)

^2^rHGP, replacement HGP

^3^TNM, tumour node metastasis

^4^CEA, carcinoembryonic antigen

*IQR, interquartile range

### Fluorine-18 fluorodeoxyglucose positron emission tomography

#### Metabolic PET activity matches histopathological findings in 130 classifiable CLM

A total of 135 CLM (69 patients) showed FDG-avid lesions concordant with MRI segment localisation and had available tissue sections; 5 CLM were non-classifiable due to absence of viable tumour on tissue sections, leaving 130 classifiable CLM for PET–HGP analyses. Figure 3 illustrates patient and CLM selection based on PET metabolic activity linked to tissue sections from the same liver segment. Details regarding CLM without PET uptake or not identified on MRI are provided in Supplementary Information 1.

#### PET parameters mean values in chemonaïve and pretreated CLM

##### Higher kurtosis in rHGP compared to eHGP in chemonaïve CLM

PET parameter averages were compared in the chemonaïve group using different reconstruction and delineation algorithms (Supplementary Table 3A). Vue Point Sharp IR + adaptive threshold algorithm showed unadjusted significant difference for kurtosis (eHGP 2.59 [1.50–3.57] vs. rHGP 3.01 [1.50–6.09]; *p* = 0.028, adjusted* p*-value = 0.99).

##### Multiple PET parameters may differentiate eHGP and rHGP in pretreated CLM

PET parameter averages were compared in the pretreated group (Supplementary Table 3B). For both VuePoint FX and VuePoint Sharp IR, unadjusted significant differences between eHGP and rHGP were observed for TLG_mean_ (fixed level threshold algorithm) and uniformity (adaptive threshold algorithm). For ‘adaptive threshold with subtraction of SUVbkg’ uniformity, kurtosis and entropy were significant for Vue Point Sharp IR only.

#### Prediction of the HGPs with PET/CT

To evaluate whether HGPs can be predicted using PET in chemonaïve CLMs, we performed univariate and multivariate logistic regression analyses with MTV, SUV_mean_, and SUV_max_ as independent variables and HGP as the dependent variable (Supplementary Tables 4A and 5A). Neither univariate nor multivariate models including only MTV, SUV_mean_ and SUV_max_ were significant. Expanded multivariate models including texture parameters and TLG_mean_ did not identify significant associations between PET parameters and HGP (Supplementary Table 6A).

To assess if HGPs can be predicted using PET in pretreated CLMs, we did univariate and multivariate logistic regression analyses with MTV, SUV_mean_, and SUV_max_ as independent variables and HGP as the dependent (Supplementary Tables 4B and 5B). Both regression analyses did not identify any significant associations with HGP. Expanded multivariate models incorporating texture parameters and TLG_mean_ yielded statistically significant associations, but extremely small odds ratios, for fixed level threshold (Supplementary Table 6B).

#### Limited association between PET parameters and overall survival

We analysed the association between PET parameters and survival in 70 patients with tumour uptake on preoperative PET/CT and available tissue sections. Two patients underwent re-resection and were included twice; their second PET/CT examination was excluded, leaving 68 patients for Cox regression analysis. Separate analyses were performed on chemonaïve and pretreated patients as previously. Among these, 40 (59%) were chemonaïve and 28 (41%) patients were pretreated. The mean time from liver surgery to death was 57 months in the chemonaïve group and 35 months in the pretreated group.

For survival analysis, the metastasis presenting the highest SUV_max_ and SUV_mean_ per patient was used. None of the parameters SUV_mean_, SUV_max_, or MTV were significant candidate parameters of survival in univariate Cox regression for either subgroup (Supplementary Table 7A and B). In multivariate Cox regression, increased SUV_max_ was associated with a trend toward significantly lower mortality risk, while increased SUV_mean_ was associated with a trend toward significantly higher mortality risk in chemonaïve patients (Table 3). Multivariate Cox regression results for both groups are provided in Supplementary Table 8A and B.

**Table 3:** Multivariate Cox regression analysis of PET parameters SUVmean, SUVmax, or MTV in chemonaïve colorectal liver metastases for associations with overall survival

Reconstruction algorithm	Delineation algorithm	PET parameter	Mortality				*N*	Event *N*
			HR	95% CI	*p*-value	Adjusted *p*-value		
Vue point FX	Adaptive threshold with subtraction of SUV_bkg_	SUV_max_	0.65	0.43–0.99	0.047	0.219	40	18
Vue point sharp IR	Fixed level threshold 50% above SUV_bkg_	SUV_max_	0.46	0.23–0.95	0.035	0.219	40	18
	Adaptive threshold with subtraction of SUV_bkg_	SUV_mean_	2.69	1.01–7.18	0.048	0.219	40	18
		SUV_max_	0.63	0.42–0.95	0.026	0.219	40	18

Only significant results presented, see Supplementary Files for the entire table (Supplementary Table 8A)

N,  number of patients, Event N,  number of subjects who had the event (death), HR, hazard ratio

#### Overall survival in histopathological growth patterns

**Fig. 4:**
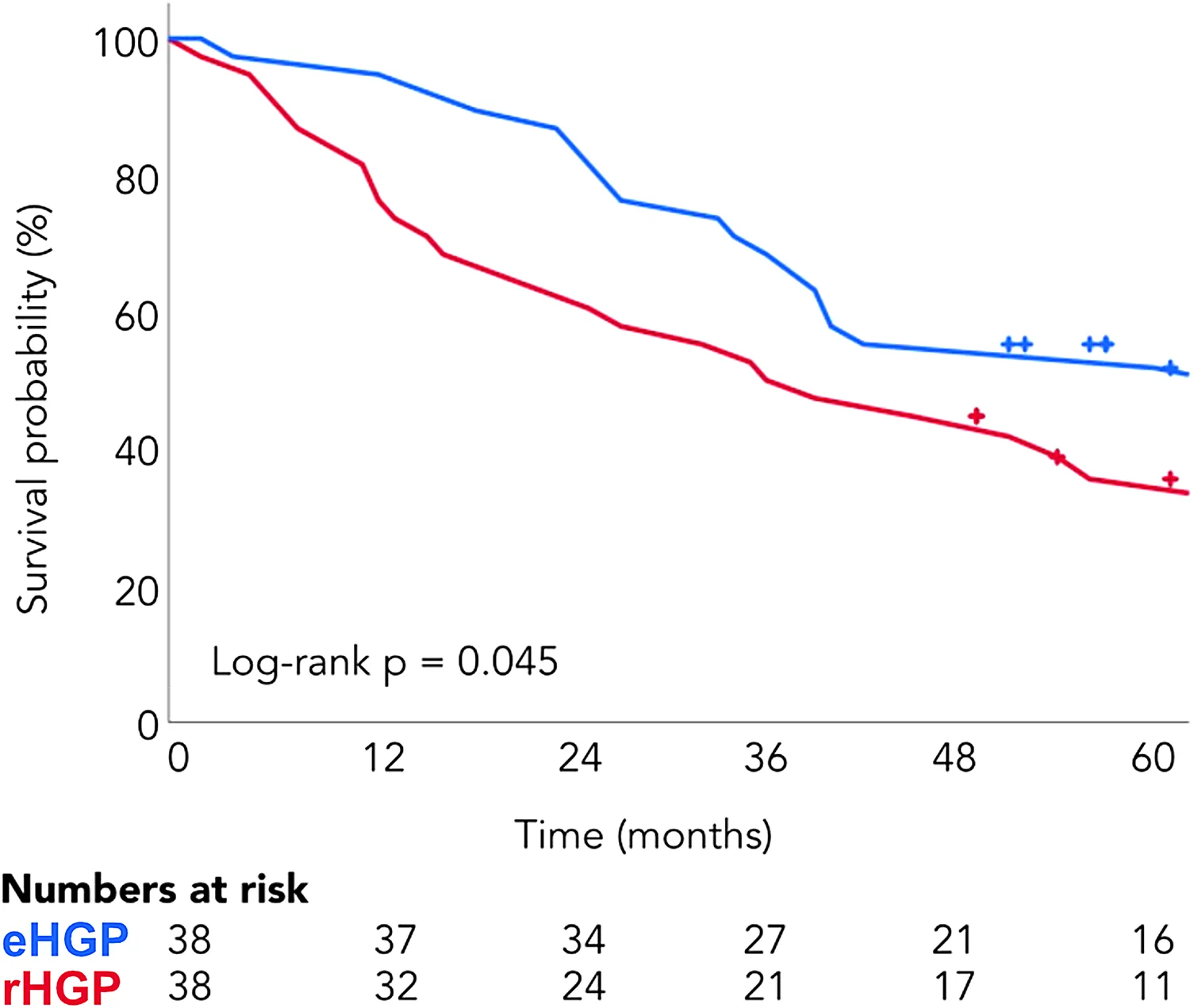
Kaplan-Meier curves presenting overall survival for all patients included divided by predominant histopathological growth pattern (HGP). eHGP, encapsulating HGP; rHGP, replacement HGP

The HGP was classifiable in 76 patients. Our study confirmed significantly better survival for patients with eHGP compared to rHGP (*p* = 0.045), see Fig. 4. This difference was not significant when chemonaïve and pretreated groups were analysed separately (Supplementary Fig. 2).

## Discussion

To our knowledge, this is the first prospective study to evaluate the potential of FDG-PET/CT for preoperative assessment of HGP in CLM. Exploratory in nature, our hypothesis-generating findings did not demonstrate reliable associations between HGP and PET parameters. Consistent with previous reports, patients with eHGP exhibited superior overall survival compared to those with rHGP [3]. A reliable imaging surrogate for HGP could improve patient stratification, guide perioperative treatment planning, and support a more individualised treatment plan. From a translational perspective, non-invasive HGP classification holds the greatest immediate potential as a tool for clinical trial stratification and preoperative risk assessment. Clinically, given that rHGP exhibits an infiltrative form associated with higher rates of R1 resections, identifying high-risk, heterogeneous lesions preoperatively could theoretically guide surgical margin strategy or prompt intensified postoperative imaging.

In chemonaïve patients, we found no significant associations between PET parameters and HGP. This contrasts with Bohlok et al. who reported higher baseline glucose uptake in non-eHGP lesions, suggesting a link between metabolic activity and HGP [21]. Several factors may account for this discrepancy. Differences in cohort composition and study design could contribute to divergent findings. HGP classification also differed where our classification relied on the predominant HGP, whereas Bohlok et al. applied a 100% cut-off. This may capture a more biologically distinct, extreme phenotype and potentially be more likely to show a measurable metabolic correlate. It is also possible that the association reported by Bohlok et al. reflects a real but modest effect that our sample was underpowered to detect. Features reflecting metabolic heterogeneity may signal rHGP, a biologically plausible finding given the distinct microenvironments of eHGP and rHGP. eHGP features a structured desmoplastic rim and abundant immune infiltration [11], supporting more uniform perfusion and metabolic behaviour. Conversely, rHGP displays an infiltrative growth and vessel co-option, which may increase spatial variation in metabolic activity. Gene expression pattern analyses confirm more aggressive proliferation and higher glucose turnover in rHGP [10]. Texture metrics may theoretically capture subtle differences in vascular organisation, stromal architecture, and metabolic gradients not reflected in SUV alone. The absence of an association in our data should not be interpreted as evidence against a true relationship between glucose metabolism and HGP. Overall, our findings do not support a role for PET-derived metabolic or textural features in predicting HGP in chemonaïve patients. They require dedicated testing in adequately powered, independent cohorts before any biological or clinical interpretation can be drawn.

In pretreated patients, multivariate analysis including texture parameters using Vue Point Sharp IR/fixed level threshold showed a significant association between metabolic and texture parameters and HGP. Although this association reached statistical significance, the corresponding odds ratios were of implausible magnitude. The small odds ratio likely reflects the limited number of events within the pretreated subgroup, raising concern for model instability and overfitting and the estimates should be interpreted with caution. We therefore do not interpret the statistical significance of these associations as evidence of a clinically meaningful or biologically interpretable effect, and the odds ratios themselves cannot be translated into an interpretable effect size. We hypothesised that MTV might outperform point measures such as SUV_max_ in the pretreated setting, since volumetric tumour burden could plausibly capture residual disease characteristics more comprehensively than a single-voxel metric. This is supported by a prior report linking pretreatment MTV to adverse outcomes in CLM [20]. Given the known biology of rHGP associated with higher proliferation and glucose turnover [10, 11], we further hypothesised that rHGP would be associated with higher MTV in pretreated patients, however this was not observed in our data. Several factors may explain this. A chemotherapy-induced shift toward eHGP with associated tumour regression has been described [27], suggesting that baseline metabolic differences between HGP subtypes may be obscured on post chemotherapy imaging. Chemotherapy-induced effects such as necrosis, fibrosis, and inflammatory infiltration may also confound both volumetric and intensity-based PET metrics, independently of HGP, further diluting any potential HGP signal. Taken together, our findings suggest that PET-derived metabolic and volumetric parameters do not reliably distinguish HGP in the pretreated setting. Our results should be regarded as exploratory and warrant larger adequately powered studies before being considered robust.

Our observations complement earlier imaging-based efforts to assess HGP in the preoperative setting. Cheng et al. developed a CT radiomics signature predictive of HGP [12], whereas our study focuses on metabolic features derived from a single PET/CT system. Combining PET with MRI may further improve classification accuracy, as MRI provides superior soft-tissue contrast and can characterise the tumour-liver interface more precisely. Exploring other molecular PET tracers for biologically targeted HGP assessment may also enhance classification. The desmoplastic rim is enriched in immune cells and cancer-associated fibroblasts, making it a potential target for immune-based tracers and fibroblast activation protein inhibitor (FAPI) PET imaging [28–30]. Such approaches may possibly better capture the stromal and immune microenvironment that differentiate eHGP from rHGP and may ultimately outperform FDG for this purpose.

Consistent with prior studies [3–5, 11], we found that eHGP was associated with superior survival following liver resection. Among chemonaïve patients, higher SUV_max_ and lower SUV_mean_ exhibited unadjusted trends toward favourable overall survival, but these signals were not adjusted for clinical, molecular and surgical factors, varied across reconstruction and delineation protocols and did not remain significant after multiple testing adjustment. The literature has yielded conflicting results regarding the prognostic value of PET-derived parameters in CLM. While a systematic review found no significant prognostic effect for SUV_max_ [20], a meta-analysis reported high SUV as a marker of poor outcome [31]. Several studies have identified volumetric measures such as TLG and MTV as relevant for overall survival [20, 32–34], liver recurrence-free survival [35], and outcomes following liver transplantation for unresectable CLM [36]. The more favourable biology of eHGP supports the hypothesis that this pattern would display lower metabolic activity and entropy. In our exploratory analysis, lower SUV_mean_ showed a weak trend toward eHGP, though results varied across threshold algorithms. Similarly, texture analysis reflecting intratumoral heterogeneity linked high entropy to adverse outcomes and may reflect a more aggressive tumour biology [36].

Differences between methods may arise from both reconstruction and delineation. VuePoint Sharp IR has higher spatial resolution than FX but is also noisier, which may reduce the sensitivity of histogram-based texture measures such as entropy and uniformity. This is in line with our finding of no significant differences between the reconstructions. Delineation methods differ in which tumour and surrounding tissue enter the VOI. A ‘fixed level threshold’ may miss low-uptake regions or include background affected by partial volume spill-out. The ‘fixed level 50% threshold above SUV_bkg_’ may reduce this problem, and the ‘adaptive threshold’ adjusts to local background and may handle heterogeneous tumours better. On visual inspection, the latter two methods delineated tumours well, whereas the ‘fixed level threshold’ appeared less suitable for small or low-uptake tumours in the liver where background activity is high. This was a qualitative observation, not supported by significant differences in our data.

Our results should be interpreted in the light of several methodological limitations. First, only FDG-avid metastases were included, limiting applicability to metabolically inactive lesions. Second, pre-imaging chemotherapy exposure may have changed tumour metabolism and reduced the discriminatory power of PET-derived features. Third, variable intervals between PET/CT acquisition and surgery may have allowed temporal changes in HGP [27]. Fourth, partial volume effects reduce SUV in small lesions [37]. Fifth, the survival models were not adjusted for established prognostic confounders in CLM. Finally, the limited sample size and large number of evaluated imaging parameters creates a high risk of model overfitting. Consequently, these results must be considered strictly hypothesis-generating and emphasise the need for external validation in larger, prospective cohorts.

The strengths of this study include it being the first prospective investigation of imaging and HGP, the use of a single-centre PET camera, and treatment planning performed within the same centre.

## Conclusion

No reliable associations were identified between FDG-PET parameters and HGP. The evaluated PET parameters therefore appear to have limited value for non-invasive HGP prediction, warranting further investigation and exploration of alternative non-invasive biomarkers including other imaging biomarkers.

## Supplementary Information

Below is the link to the electronic supplementary material.

Supplementary Material 1

## Data Availability

Data can be made available after reasonable request to the corresponding author.
