# Supplementary material for: Preoperative classification of histopathological growth patterns in colorectal liver metastases using prospective 18 F-FDG PET/CT
Source: Clin Exp Metastasis. 2026 Nov 7;43(6):63. doi: 10.1007/s10585-026-10441-x (PMC13743569; doi:10.1007/s10585-026-10441-x)
Supplement: Supplementary file 1 [file 10585_2026_10441_MOESM1_ESM.docx]

Supplementary Files/Information

**Preoperative classification of Histopathological Growth Patterns in Colorectal Liver Metastases using prospective ^18^F-FDG PET/CT**

*Clinical & Experimental Metastasis*

J. Jonsson (JJ)^a^, A. Ling (AL)^b^, J. Axelsson (JA)^c^, Mathias Lundin^a,d^, P. Vermeulen (PV)^e^, K. Riklund (KR)^f^ and H. Nyström (HN)^a,g^

^a^Department of Diagnostics and Intervention, Surgery, Umeå University, Sweden

^b^Department of Medical Biosciences, Pathology, Umeå University, Sweden

^c^Department of Diagnostics and Intervention, Radiation Physics, Umeå University, Sweden

^d^Department of Statistics, Umeå School of Business, Economics and Statistics (USBE), Statistics Umeå University, Umeå, Sweden

^e^Translational Cancer Research Unit, Oncology Center Antwerp, Ziekenhuis aan de Stroom, Wilrijk, Antwerp, Belgium

^f^Department of Diagnostics and Intervention, Diagnostic Radiology, Umeå University, Sweden

^g^Wallenberg Centre for Molecular Medicine, Umeå University, Sweden

**Author responsible for correspondence:**

Name: Josefin Jonsson

E-mail address: [josefin.jonsson@umu.se](mailto:josefin.jonsson@umu.se)

**Supplementary Fig. 1 Flowchart of inclusion describing recruitment of patients**
CLM = colorectal liver metastases

**Supplementary Table** **1.** Description of positron emission tomography (PET) parameters extracted from each metastasis/volume of interest (VOI) from the standardised uptake (SUV) images.

| **PET parameter** |  |
| --- | --- |
| SUV | Standardised uptake value, PET measured radioactive concentration relative to injected distributed in the whole body |
| SUV_mean_ | (g/mL), average SUV within a VOI |
| SUV_max_ | (g/mL), SUV of the pixel within the VOI with the highest SUV |
| MTV | (cm^3^), metabolic tumour volume, a value of the metabolically active volume of the VOI. |
| TLG_mean_ | Total lesion glycolysis mean = SUV_mean_ * MTV |
| **PET texture parameters** | |
| Standard deviation (SD) | Standard deviation of SUV values within a VOI |
| Skewness | A measure describing the texture of SUV values within a VOI. Indicates how symmetric the distribution of the voxel values is |
| Kurtosis | A measure describing the texture of SUV values within a VOI. Indicates if there are voxels that are outliers relative the distribution |
| Uniformity | A measure describing the texture of SUV values within a VOI. Gives a number to how uniform the data within the VOI is. |
| Entropy | A measure describing the texture of SUV values within a VOI. Gives a measure about the randomness of the data within the VOI. |

**Supplementary Information 1.** Additional information about colorectal liver metastases (CLM) excluded from PET analysis.

To correlate PET parameters with HGP, we only included lesions with HGP classification and measurable uptake on PET/CT in the subsequent regression analyses. Some patients exhibited mixed nuclear medicine imaging with CLMs displaying both uptake and no uptake on PET/CT. In total, 58 CLMs from 24 patients showed no uptake on PET/CT. Among these, seven patients (17 CLMs) demonstrated no uptake in any metastasis, five of whom had received pre-imaging chemotherapy. Overall, pre-imaging chemotherapy was administered to 44 (76%) CLMs without metabolic activity.

Fifteen (26%) CLM without metabolic activity on PET were predominant replacement HGP (rHGP) ≥50%, and ten of them received pre-imaging chemotherapy; 31 (53%) CLM were predominant encapsulating HGP (eHGP), and 25 eHGP CLM received chemotherapy before imaging. Twelve (21%) CLMs in eight patients were non-classifiable, of which eight CLMs and seven patients had received pre-imaging chemotherapy

Four metastases in two patients included for HGP classification were not identified on MRI and thus excluded from further analysis of PET data. In one patient three CLM were classified as eHGP ≥50% (one CLM with 100% eHGP) and no pre-imaging chemotherapy was administered. The second patient had received pre-imaging chemotherapy and had one non-classifiable CLM.

| **Supplementary Table 2. Characteristics of included patients comparing chemonaïve and pretreated patients.** | | | |
| --- | --- | --- | --- |
|  | **Chemonaïve** (n=44) | **Pretreated** (n=34) | **p-value** |
| Age at surgery, years (median, [IQR^1^]) | 66.5 (58.5-74) | 66 (57-70) | 0.248 |
| Gender, Male:Female (n, [%]) | 28 (64%): 16 (36%) | 23 (68%): 11 (32%) | 0.712 |
| **TNM**^2^**-stage at CRC**^3^ **diagnosis** | | | |
| I (n, [%]) | 4 (9%) | 0 (0%) | <0.001 |
| II (n, [%]) | 6 (13.5%) | 0 (0%) |  |
| III (n, [%]) | 14 (32%) | 5 (15%) |  |
| IV (n, [%]) | 20 (45.5%) | 29 (85%) |  |
| **Extrahepatic disease (EHD)** | | | |
| Existing EHD known at time of surgery (n, [%]) | 8 (18%) | 7 (21%) | 0.789 |
| Previous resected EHD (n, [%]) | 1 (2%) | 2 (6%) | 0.577 |
| Synchronous resection of EHD (n, [%]) | 5 (11%) | 5 (15%) | 0.740 |
| **CEA**^4^**-value** |  |  |  |
| Preoperative CEA-value, µg/L (median, [IQR]) | 5.65 (2.60-40) | 9.15 (3.08-37.25) | 0.389 |
| **Colorectal liver metastases** | | | |
| Tumour number (median, [IQR]) | 2 (1-3) | 6 (3-11.5) | <0.001 |
| Tumour size in cm (median, [IQR]) | 3 (1.7-4.4) | 3 (2-3.5) | 0.844 |
| *Preoperative chemotherapy in relation to liver resection* | | | |
| No chemotherapy (n, [%]) | 44 (100%) | 0 (100%) | <0.01 |
| Neoadjuvant (n, [%]) | 0 (0%) | 22 (65%) |  |
| Downsize/conversion therapy (n, [%]) | 0 (0%) | 12 (35%) |  |
| *Postoperative chemotherapy in relation to liver resection* | | | |
| No adjuvant chemotherapy (n, [%]) | 22 (50%) | 14 (39%) | 0.22 |
| Adjuvant (n, [%]) | 21 (48%) | 15 (44%) |  |
| Palliative chemotherapy (n, [%]) | 1 (2%) | 4 (12%) |  |
| Missing information (n, [%]) | 0 (0%) | 1 (3%) |  |
| **Repeated resections** |  |  |  |
| Repeat resection after this resection (n, [%]) | 4 (9%) | 8 (24%) | 0.088 |
| Previous resection (n, [%]) | 3 (7%) | 5 (15%) |  |
| ^1^IQR = interquartile range  ^2^TNM = tumour node metastasis staging system  ^3^CRC = colorectal cancer  ^4^CEA = carcinoembryonic antigen | | | |

**Supplementary Table 3A + 3B. Comparison of PET parameters between replacement (rHGP) and encapsulating (eHGP) colorectal liver metastases A) without oncological treatment prior imaging and liver surgery and B) with oncological treatment prior imaging and liver surgery.** Bold numbers with asterisk are statistically significant.

| **Supplementary Table 3A. Chemonaïve colorectal liver metastases** | | | | | | |
| --- | --- | --- | --- | --- | --- | --- |
| **Reconstruction algorithm** | **Delineation algorithm** | **PET parameter** | **p-value** | **Adjusted**  **p-value** | **eHGP ≥50%** (n) | **rHGP ≥50%** (n) |
| **Vue Point FX** | Fixed level treshold | SUV_mean_ | 0.956 | 0.99 | 25 | 40 |
|  |  | SUV_max_ | 0.807 | 0.99 |  |  |
|  |  | MTV | 0.093 | 0.99 |  |  |
|  |  | TLG_mean_ | 0.278 | 0.99 |  |  |
|  |  | SD | 0.798 | 0.99 |  |  |
|  |  | Skewness | 0.133 | 0.99 |  |  |
|  |  | Kurtosis | 0.400 | 0.99 |  |  |
|  |  | Uniformity | 0.222 | 0.99 |  |  |
|  |  | Entropy | 0.323 | 0.99 |  |  |
|  | Adaptive treshold | SUV_mean_ | 0.539 | 0.99 | 25 | 40 |
|  |  | SUV_max_ | 0.976 | 0.99 |  |  |
|  |  | MTV | 0.574 | 0.99 |  |  |
|  |  | TLG_mean_ | 0.688 | 0.99 |  |  |
|  |  | SD | 0.542 | 0.99 |  |  |
|  |  | Skewness | 0.440 | 0.99 |  |  |
|  |  | Kurtosis | 0.137 | 0.99 |  |  |
|  |  | Uniformity | 0.346 | 0.99 |  |  |
|  |  | Entropy | 0.677 | 0.99 |  |  |
|  | Fixed level treshold 50% above SUV_bkg_ | SUV_mean_ | 0.935 | 0.99 | 25 | 40 |
|  |  | SUV_max_ | 0.842 | 0.99 |  |  |
|  |  | MTV | 0.216 | 0.99 |  |  |
|  |  | TLG_mean_ | 0.456 | 0.99 |  |  |
|  |  | SD | 0.666 | 0.99 |  |  |
|  |  | Skewness | 0.967 | 0.99 |  |  |
|  |  | Kurtosis | 0.582 | 0.99 |  |  |
|  |  | Uniformity | 0.967 | 0.99 |  |  |
|  |  | Entropy | 0.990 | 0.99 |  |  |
|  | Adaptive treshold with subtraction of SUV_bkg_ | SUV_mean_ | 0.838 | 0.99 | 25 | 40 |
|  |  | SUV_max_ | 0.601 | 0.99 |  |  |
|  |  | MTV | 0.401 | 0.99 |  |  |
|  |  | TLG_mean_ | 0.656 | 0.99 |  |  |
|  |  | SD | 0.436 | 0.99 |  |  |
|  |  | Skewness | 0.467 | 0.99 |  |  |
|  |  | Kurtosis | 0.851 | 0.99 |  |  |
|  |  | Uniformity | 0.414 | 0.99 |  |  |
|  |  | Entropy | 0.561 | 0.99 |  |  |
| **Vue Point Sharp IR** | Fixed level treshold | SUV_mean_ | 0.953 | 0.99 | 25 | 40 |
|  |  | SUV_max_ | 0.972 | 0.99 |  |  |
|  |  | MTV | 0.095 | 0.99 |  |  |
|  |  | TLG_mean_ | 0.298 | 0.99 |  |  |
|  |  | SD | 0.847 | 0.99 |  |  |
|  |  | Skewness | 0.377 | 0.99 |  |  |
|  |  | Kurtosis | 0.582 | 0.99 |  |  |
|  |  | Uniformity | 0.308 | 0.99 |  |  |
|  |  | Entropy | 0.560 | 0.99 |  |  |
|  | Adaptive treshold | SUV_mean_ | 0.720 | 0.99 | 25 | 40 |
|  |  | SUV_max_ | 0.743 | 0.99 |  |  |
|  |  | MTV | 0.435 | 0.99 |  |  |
|  |  | TLG_mean_ | 0.583 | 0.99 |  |  |
|  |  | SD | 0.724 | 0.99 |  |  |
|  |  | Skewness | 0.328 | 0.99 |  |  |
|  |  | **Kurtosis** | **0.028*** | 0.99 |  |  |
|  |  | Uniformity | 0.343 | 0.99 |  |  |
|  |  | Entropy | 0.744 | 0.99 |  |  |
|  | Fixed level treshold 50% above SUV_bkg_ | SUV_mean_ | 0.911 | 0.99 | 25 | 40 |
|  |  | SUV_max_ | 0.980 | 0.99 |  |  |
|  |  | MTV | 0.213 | 0.99 |  |  |
|  |  | TLG_mean_ | 0.470 | 0.99 |  |  |
|  |  | SD | 0.833 | 0.99 |  |  |
|  |  | Skewness | 0.187 | 0.99 |  |  |
|  |  | Kurtosis | 0.058 | 0.99 |  |  |
|  |  | Uniformity | 0.803 | 0.99 |  |  |
|  |  | Entropy | 0.717 | 0.99 |  |  |
|  | Adaptive treshold with subtraction of SUV_bkg_ | SUV_mean_ | 0.587 | 0.99 | 25 | 40 |
|  |  | SUV_max_ | 0.846 | 0.99 |  |  |
|  |  | MTV | 0.429 | 0.99 |  |  |
|  |  | TLG_mean_ | 0.667 | 0.99 |  |  |
|  |  | SD | 0.465 | 0.99 |  |  |
|  |  | Skewness | 0.411 | 0.99 |  |  |
|  |  | Kurtosis | 0.914 | 0.99 |  |  |
|  |  | Uniformity | 0.616 | 0.99 |  |  |
|  |  | Entropy | 0.834 | 0.99 |  |  |

SUV_bkg_ = standardised uptake value of the liver background

| **Supplementary Table 3B. Pretreated colorectal liver metastases** | | | | | | |
| --- | --- | --- | --- | --- | --- | --- |
| **Reconstruction algorithm** | **Delineation algorithm** | **PET parameter** | **p-value** | **Adjusted  p-value** | **eHGP ≥50% (n)** | **rHGP ≥50% (n)** |
| **Vue Point FX** | Fixed level threshold | SUV_mean_ | 0.445 | 0.877 | 20 | 44 |
|  |  | SUV_max_ | 0.368 | 0.803 |  |  |
|  |  | MTV | 0.153 | 0.689 |  |  |
|  |  | **TLG_mean_** | **0.043*** | 0.442 |  |  |
|  |  | SD | 0.365 | 0.803 |  |  |
|  |  | Skewness | 0.179 | 0.701 |  |  |
|  |  | Kurtosis | 0.796 | 0.911 |  |  |
|  |  | Uniformity | 0.516 | 0.877 |  |  |
|  |  | Entropy | 0.664 | 0.877 |  |  |
|  | Adaptive threshold | SUV_mean_ | 0.239 | 0.711 | 19 | 44 |
|  |  | SUV_max_ | 0.692 | 0.877 |  |  |
|  |  | MTV | 0.527 | 0.877 |  |  |
|  |  | TLG_mean_ | 0.520 | 0.877 |  |  |
|  |  | SD | 0.755 | 0.911 |  |  |
|  |  | Skewness | 0.309 | 0.778 |  |  |
|  |  | Kurtosis | 0.247 | 0.711 |  |  |
|  |  | **Uniformity** | **0.009*** | 0.408 |  |  |
|  |  | Entropy | 0.060 | 0.48 |  |  |
|  | Fixed level threshold 50% above SUV_bkg_ | SUV_mean_ | 0.662 | 0.877 | 18 | 44 |
|  |  | SUV_max_ | 0.567 | 0.877 |  |  |
|  |  | MTV | 0.101 | 0.55 |  |  |
|  |  | TLG_mean_ | 0.214 | 0.701 |  |  |
|  |  | SD | 0.538 | 0.877 |  |  |
|  |  | Skewness | 0.617 | 0.877 |  |  |
|  |  | Kurtosis | 0.925 | 0.965 |  |  |
|  |  | Uniformity | 0.638 | 0.877 |  |  |
|  |  | Entropy | 0.265 | 0.734 |  |  |
|  | Adaptive threshold with subtraction of SUV_bkg_ | SUV_mean_ | 0.498 | 0.877 | 18 | 44 |
|  |  | SUV_max_ | 0.763 | 0.911 |  |  |
|  |  | MTV | 0.173 | 0.701 |  |  |
|  |  | TLG_mean_ | 0.290 | 0.773 |  |  |
|  |  | SD | 0.689 | 0.877 |  |  |
|  |  | Skewness | 0.656 | 0.877 |  |  |
|  |  | Kurtosis | 0.616 | 0.877 |  |  |
|  |  | Uniformity | 0.087 | 0.55 |  |  |
|  |  | Entropy | 0.107 | 0.55 |  |  |
| **Vue Point Sharp IR** | Fixed level threshold | SUV_mean_ | 0.481 | 0.877 | 20 | 44 |
|  |  | SUV_max_ | 0.694 | 0.689 |  |  |
|  |  | MTV | 0.153 | 0.689 |  |  |
|  |  | **TLG_mean_** | **0.042*** | 0.442 |  |  |
|  |  | SD | 0.622 | 0.877 |  |  |
|  |  | Skewness | 0.970 | 0.97 |  |  |
|  |  | Kurtosis | 0.418 | 0.877 |  |  |
|  |  | Uniformity | 0.799 | 0.911 |  |  |
|  |  | Entropy | 0.872 | 0.951 |  |  |
|  | Adaptive threshold | SUV_mean_ | 0.368 | 0.803 | 19 | 44 |
|  |  | SUV_max_ | 0.797 | 0.911 |  |  |
|  |  | MTV | 0.741 | 0.911 |  |  |
|  |  | TLG_mean_ | 0.5 | 0.877 |  |  |
|  |  | SD | 0.962 | 0.97 |  |  |
|  |  | Skewness | 0.872 | 0.951 |  |  |
|  |  | Kurtosis | 0.488 | 0.877 |  |  |
|  |  | **Uniformity** | **0.012*** | 0.408 |  |  |
|  |  | Entropy | 0.099 | 0.55 |  |  |
|  | Fixed level threshold 50% above SUV_bkg_ | SUV_mean_ | 0.941 | 0.968 | 18 | 44 |
|  |  | SUV_max_ | 0.924 | 0.965 |  |  |
|  |  | MTV | 0.101 | 0.55 |  |  |
|  |  | TLG_mean_ | 0.222 | 0.701 |  |  |
|  |  | SD | 0.645 | 0.877 |  |  |
|  |  | Skewness | 0.222 | 0.701 |  |  |
|  |  | Kurtosis | 0.673 | 0.877 |  |  |
|  |  | Uniformity | 0.454 | 0.877 |  |  |
|  |  | Entropy | 0.224 | 0.701 |  |  |
|  | Adaptive threshold with subtraction of SUV_bkg_ | SUV_mean_ | 0.324 | 0.778 | 18 | 44 |
|  |  | SUV_max_ | 0.924 | 0.965 |  |  |
|  |  | MTV | 0.22 | 0.701 |  |  |
|  |  | TLG_mean_ | 0.315 | 0.778 |  |  |
|  |  | SD | 0.81 | 0.911 |  |  |
|  |  | Skewness | 0.058 | 0.48 |  |  |
|  |  | **Kurtosis** | **0.025*** | 0.442 |  |  |
|  |  | **Uniformity** | **0.017*** | 0.408 |  |  |
|  |  | **Entropy** | **0.041*** | 0.442 |  |  |

SUV_bkg_ = standardised uptake value of the liver background

**LOGISTIC REGRESSION**

**Supplementary Table 4A + 4B. Univariate logistic regression of PET parameters in colorectal liver metastases A) without oncological treatment prior imaging and liver surgery and B) with oncological treatment prior imaging and liver surgery, for associations with HGP.** Dependent variable = encapsulating HGP.

| **Supplementary Table 4A. Chemonaïve colorectal liver metastases** | | | | | |  |  |
| --- | --- | --- | --- | --- | --- | --- | --- |
| **Reconstruction algorithm** | **Delineation algorithm** | **PET parameter** | **Odds** | | | **CLM** | **CLM missing** |
|  |  |  | **Odds ratio** | **95% CI** | **p-value** |  |  |
| **Vue Point FX** | Fixed level threshold | SUV_mean_ | 0.86 | 0.43-1.72 | 0.675 | 65 | 2 |
|  |  | SUV_max_ | 0.94 | 0.63-1.4 | 0.749 | 65 | 2 |
|  |  | MTV | 0.94 | 0.85-1.03 | 0.154 | 65 | 2 |
|  | Adaptive threshold | SUV_mean_ | 0.65 | 0.21-1.99 | 0.445 | 63 | 4 |
|  |  | SUV_max_ | 0.93 | 0.62-1.38 | 0.702 | 63 | 4 |
|  |  | MTV | 0.97 | 0.89-1.05 | 0.418 | 65 | 2 |
|  | Fixed level threshold 50% above SUV_bkg_ | SUV_mean_ | 0.83 | 0.40-1.73 | 0.616 | 65 | 2 |
|  |  | SUV_max_ | 0.92 | 0.61-1.39 | 0.704 | 65 | 2 |
|  |  | MTV | 0.92 | 0.80-1.04 | 0.260 | 65 | 2 |
|  | Adaptive threshold with subtraction of SUV_bkg_ | SUV_mean_ | 0.74 | 0.26-2.05 | 0.556 | 64 | 3 |
|  |  | SUV_max_ | 0.98 | 0.68-1.41 | 0.908 | 64 | 3 |
|  |  | MTV | 0.96 | 0.89-1.04 | 0.305 | 65 | 2 |
| **Vue Point Sharp IR** | Fixed level threshold | SUV_mean_ | 0.86 | 0.45-1.62 | 0.636 | 65 | 2 |
|  |  | SUV_max_ | 0.88 | 0.61-1.29 | 0.523 | 65 | 2 |
|  |  | MTV | 0.94 | 0.86-1.02 | 0.156 | 65 | 2 |
|  | Adaptive threshold | SUV_mean_ | 0.76 | 0.27-2.10 | 0.596 | 63 | 4 |
|  |  | SUV_max_ | 0.85 | 0.57-1.28 | 0.444 | 63 | 4 |
|  |  | MTV | 0.95 | 0.87-1.05 | 0.321 | 65 | 2 |
|  | Fixed level treshold 50% above SUV_bkg_ | SUV_mean_ | 0.83 | 0.42-1.64 | 0.598 | 65 | 2 |
|  |  | SUV_max_ | 0.87 | 0.59-1.28 | 0.480 | 65 | 2 |
|  |  | MTV | 0.92 | 0.79-1.06 | 0.258 | 65 | 2 |
|  | Adaptive treshold with subtraction of SUV_bkg_ | SUV_mean_ | 0.56 | 0.14-2.25 | 0.412 | 64 | 3 |
|  |  | SUV_max_ | 0.90 | 0.26-1.30 | 0.559 | 64 | 3 |
|  |  | MTV | 0.96 | 0.89-1.04 | 0.319 | 65 | 2 |

CLM = number of colorectal liver metastases analysed, CLM missing = metastases with missing PET parameter for specific delineation algorithm

| **Supplementary Table 4B. Pretreated colorectal liver metastases** | | | | | |  |  |
| --- | --- | --- | --- | --- | --- | --- | --- |
| **Reconstruction algorithm** | **Delineation algorithm** | **PET parameter** | **Odds** | | | **CLM** | **CLM missing** |
|  |  |  | **Odds ratio** | **95% CI** | **p-value** |  |  |
| **Vue Point FX** | Fixed level threshold | SUV_mean_ | 0.53 | 0.22-1.25 | 0.146 | 64 | 5 |
|  |  | SUV_max_ | 0.68 | 0.42-1.09 | 0.110 | 64 | 5 |
|  |  | MTV | 0.97 | 9.85-1.10 | 0.635 | 64 | 5 |
|  | Adaptive threshold | SUV_mean_ | 0.29 | 0.08-1.06 | 0.062 | 62 | 6 |
|  |  | SUV_max_ | 0.68 | 0.42-1.11 | 0.125 | 62 | 6 |
|  |  | MTV | 0.94 | 0.76-1.15 | 0.528 | 63 | 5 |
|  | Fixed level threshold 50% above SUV_bkg_ | SUV_mean_ | 0.54 | 0.24-1.19 | 0.125 | 62 | 5 |
|  |  | SUV_max_ | 0.70 | 0.44-1.12 | 0.139 | 62 | 5 |
|  |  | MTV | 0.73 | 0.45-1.18 | 0.201 | 62 | 5 |
|  | Adaptive threshold with subtraction of SUV_bkg_ | SUV_mean_ | 0.41 | 0.11-1.48 | 0.172 | 62 | 5 |
|  |  | SUV_max_ | 0.73 | 0.46-1.15 | 0.177 | 62 | 5 |
|  |  | MTV | 0.89 | 0.75-1.06 | 0.204 | 62 | 5 |
| **Vue Point Sharp IR** | Fixed level threshold | SUV_mean_ | 0.55 | 0.25-1.23 | 0.145 | 64 | 5 |
|  |  | SUV_max_ | 0.76 | 0.52-1.11 | 0.153 | 64 | 5 |
|  |  | MTV | 0.97 | 0.85-1.10 | 0.635 | 64 | 5 |
|  | Adaptive threshold | SUV_mean_ | 0.36 | 0.11-1.22 | 0.102 | 62 | 6 |
|  |  | SUV_max_ | 0.74 | 0.50-1.11 | 0.144 | 62 | 6 |
|  |  | MTV | 0.94 | 0.77-1.14 | 0.531 | 63 | 5 |
|  | Fixed level treshold 50% above SUV_bkg_ | SUV_mean_ | 0.59 | 0.29-1.20 | 0.146 | 62 | 5 |
|  |  | SUV_max_ | 0.79 | 0.55-1.14 | 0.207 | 62 | 5 |
|  |  | MTV | 0.73 | 0.45-1.18 | 0.201 | 62 | 5 |
|  | Adaptive treshold with subtraction of SUV_bkg_ | SUV_mean_ | 0.36 | 0.10-1.27 | 0.111 | 62 | 5 |
|  |  | SUV_max_ | 0.79 | 0.55-1.14 | 0.207 | 62 | 5 |
|  |  | MTV | 0.91 | 0.77-1.08 | 0.288 | 62 | 5 |

CLM = number of colorectal liver metastases analysed, CLM missing = metastases with missing PET parameter for specific delineation algorithm

**Supplementary Table 5A + 5B. Multivariate logistic regression of PET parameters in colorectal liver metastases A) without oncological treatment prior imaging and liver surgery and B) A) with oncological treatment prior imaging and liver surgery, for associations with HGP.** Dependent variable = encapsulating HGP (eHGP).

**Supplementary Table 5A. Chemonaïve colorectal liver metastases**

| **Reconstruction algorithm** | **Delineation algorithm** | **PET parameter** | **Odds** | | | | **CLM** | **CLM missing** |
| --- | --- | --- | --- | --- | --- | --- | --- | --- |
|  |  |  | **Odds ratio** | **95% CI** | **p-value** | **Adjusted p-value** |  |  |
| **Vue Point FX** | Fixed level threshold | SUV_mean_ | 0.22 | 0.00-10.2 | 0.438 | 0.438 | 65 | 2 |
|  |  | SUV_max_ | 2.65 | 0.26-27.5 | 0.413 | 0.438 |  |  |
|  |  | MTV | 0.93 | 0.85-1.03 | 0.159 | 0.438 |  |  |
|  | Adaptive threshold | SUV_mean_ | 0.17 | 0.01-3.60 | 0.257 | 0.386 | 63 | 4 |
|  |  | SUV_max_ | 1.92 | 0.64-5.77 | 0.243 | 0.386 |  |  |
|  |  | MTV | 0.98 | 0.90-1.08 | 0.698 | 0.698 |  |  |
|  | Fixed level threshold 50% above SUV_bkg_ | SUV_mean_ | 0.00 | 0.00-1428 | 0.328 | 0.328 | 65 | 2 |
|  |  | SUV_max_ | 88.5 | 0.02-511538 | 0.310 | 0.328 |  |  |
|  |  | MTV | 0.85 | 0.65-1.13 | 0.265 | 0.328 |  |  |
|  | Adaptive threshold with subtraction of SUV_bkg_ | SUV_mean_ | 0.45 | 0.06-3.13 | 0.419 | 0.419 | 64 | 3 |
|  |  | SUV_max_ | 1.51 | 0.70-3.22 | 0.293 | 0.419 |  |  |
|  |  | MTV | 0.97 | 0.90-1.04 | 0.357 | 0.419 |  |  |
| **Vue Point Sharp IR** | Fixed level threshold | SUV_mean_ | 1.79 | 0.19-17.2 | 0.613 | 0.613 | 65 | 2 |
|  |  | SUV_max_ | 0.72 | 0.20-2.51 | 0.601 | 0.613 |  |  |
|  |  | MTV | 0.94 | 0.86-1.03 | 0.165 | 0.494 |  |  |
|  | Adaptive threshold | SUV_mean_ | 8.69 | 0.04-1833 | 0.428 | 0.643 | 63 | 4 |
|  |  | SUV_max_ | 0.37 | 0.06-2.27 | 0.282 | 0.643 |  |  |
|  |  | MTV | 0.99 | 0.83-1.19 | 0.937 | 0.937 |  |  |
|  | Fixed level treshold 50% above SUV_bkg_ | SUV_mean_ | 3.31 | 0.06-182 | 0.558 | 0.563 | 65 | 2 |
|  |  | SUV_max_ | 0.51 | 0.05-5.00 | 0.563 | 0.563 |  |  |
|  |  | MTV | 0.93 | 0.79-1.10 | 0.393 | 0.563 |  |  |
|  | Adaptive treshold with subtraction of SUV_bkg_ | SUV_mean_ | 0.44 | 0.05-4.15 | 0.477 | 0.654 | 64 | 3 |
|  |  | SUV_max_ | 1.20 | 0.65-2.23 | 0.561 | 0.654 |  |  |
|  |  | MTV | 0.98 | 0.91-1.06 | 0.654 | 0.654 |  |  |

CLM = number of colorectal liver metastases analysed, CLM missing = metastases with missing PET parameter for specific delineation algorithm

**Supplementary Table 5B. Pretreated colorectal liver metastases**

| **Reconstruction algorithm** | **Delineation algorithm** | **PET parameter** | **Odds** | | | | **CLM** | **CLM missing** |
| --- | --- | --- | --- | --- | --- | --- | --- | --- |
|  |  |  | **Odds ratio** | **95% CI** | **p-value** | **Adjusted p-value** |  |  |
| **Vue Point FX** | Fixed level threshold | SUV_mean_ | 1.14 | 0.08-1.12 | 0.632 | 0.923 | 64 | 5 |
|  |  | SUV_max_ | 0.63 | 0.12-3.40 | 0.593 | 0.923 |  |  |
|  |  | MTV | 0.96 | 0.83-1.12 | 0.632 | 0.923 |  |  |
|  | Adaptive threshold | SUV_mean_ | 0.15 | 0.01-1.77 | 0.132 | 0.395 | 62 | 6 |
|  |  | SUV_max_ | 1.37 | 0.46-4.04 | 0.573 | 0.860 |  |  |
|  |  | MTV | 1.00 | 0.78-1.28 | 0.981 | 0.981 |  |  |
|  | Fixed level threshold 50% above SUV_bkg_ | SUV_mean_ | 0.01 | 0.00-220 | 0.390 | 0.435 | 62 | 5 |
|  |  | SUV_max_ | 9.89 | 0.03-3107 | 0.435 | 0.435 |  |  |
|  |  | MTV | 0.81 | 0.49-1.32 | 0.395 | 0.435 |  |  |
|  | Adaptive threshold with subtraction of SUV_bkg_ | SUV_mean_ | 0.55 | 0.05-7.14 | 0.651 | 0.922 | 62 | 5 |
|  |  | SUV_max_ | 1.05 | 0.38-2.96 | 0.922 | 0.922 |  |  |
|  |  | MTV | 0.92 | 0.73-1.17 | 0.504 | 0.922 |  |  |
| **Vue Point Sharp IR** | Fixed level threshold | SUV_mean_ | 0.35 | 0.03-3.69 | 0.381 | 0.663 | 64 | 5 |
|  |  | SUV_max_ | 1.29 | 0.41-4.03 | 0.663 | 0.663 |  |  |
|  |  | MTV | 0.96 | 0.81-1.12 | 0.582 | 0.663 |  |  |
|  | Adaptive threshold | SUV_mean_ | 0.39 | 0.06-2.36 | 0.303 | 0.910 | 62 | 6 |
|  |  | SUV_max_ | 0.98 | 0.49-1.96 | 0.957 | 0.957 |  |  |
|  |  | MTV | 0.98 | 0.76-1.28 | 0.909 | 0.957 |  |  |
|  | Fixed level treshold 50% above SUV_bkg_ | SUV_mean_ | 0.04 | 0.00-1.80 | 0.097 | 0.167 | 62 | 5 |
|  |  | SUV_max_ | 5.19 | 0.68-39.5 | 0.112 | 0.167 |  |  |
|  |  | MTV | 0.73 | 0.44-1.21 | 0.226 | 0.226 |  |  |
|  | Adaptive treshold with subtraction of SUV_bkg_ | SUV_mean_ | 0.30 | 0.04-2.06 | 0.219 | 0.655 | 62 | 5 |
|  |  | SUV_max_ | 1.16 | 0.64-2.11 | 0.618 | 0.655 |  |  |
|  |  | MTV | 0.96 | 0.78-1.17 | 0.655 | 0.655 |  |  |

CLM = number of colorectal liver metastases analysed, CLM missing = metastases with missing PET parameter for specific delineation algorithm

**Supplementary Table 6A + 6B. Multivariate logistic regression of PET parameters, including texture parameters, in colorectal liver metastases A) without oncological treatment prior imaging and liver surgery and B) with oncological treatment prior imaging and liver surgery, for associations with HGP.** Dependent variable = encapsulating HGP (eHGP).

**Supplementary Table 6A. Chemonaïve colorectal liver metastases**

| **Reconstruction algorithm** | **Delineation algorithm** | **PET parameter** | **Odds** | | | | **CLM** | **CLM missing** |
| --- | --- | --- | --- | --- | --- | --- | --- | --- |
|  |  |  | **Odds ratio** | **95% CI** | **p-value** | **Adjusted p-value** |  |  |
| **Vue Point FX** | Fixed level threshold | SUV_mean_ | 9.25 x 10^2^ | 9.31 x 10^-8^-9.19 x 10^12^ | 0.561 | 0.678 | 65 | 2 |
|  |  | SUV_max_ | 0.432 | 2.11 x 10^-4^-8.86 x 10^2^ | 0.829 | 0.829 |  |  |
|  |  | MTV | 0.769 | 0.514-1.15 | 0.201 | 0.678 |  |  |
|  |  | SD | 1.20 x 10^-10^ | 3.79 x 10^-35^-3.82 x 10^14^ | 0.427 | 0.678 |  |  |
|  |  | Skewness | 5.58 x 10^3^ | 1.69 x 10^-7^-1.84 x 10^14^ | 0.485 | 0.678 |  |  |
|  |  | Kurtosis | 0.115 | 5.21 x 10^-5^-2.55 x 10^2^ | 0.582 | 0.678 |  |  |
|  |  | Log(uniformity) | 2.56 x 10^5^ | 1.10 x 10^5^-5.97 x 10^25^ | 0.603 | 0.678 |  |  |
|  |  | Entropy | 2.30 x 10^5^ | 2.36 x 10^-11^ -2.23 x 10^21^ | 0.511 | 0.678 |  |  |
|  |  | TLG_mean_ | 1.02 | 0.976-1.07 | 0.372 | 0.678 |  |  |
|  | Adaptive threshold | SUV_mean_ | 1.18 × 10⁻² | 2.01 × 10⁻⁴-6.94 × 10⁻¹ | 0.033 | 0.198 | 62 | 5 |
|  |  | SUV_max_ | 23.7 | 0.706-796 | 0.077 | 0.198 |  |  |
|  |  | MTV | 0.654 | 0.394-1.09 | 0.100 | 0.198 |  |  |
|  |  | SD | 6.95 × 10⁻⁴ | 4.01 × 10⁻⁸-12.1 | 0.144 | 0.198 |  |  |
|  |  | Skewness | 139 | 4.53 × 10⁻³-4.28 × 10⁶ | 0.349 | 0.349 |  |  |
|  |  | Kurtosis | 7.46 × 10⁻³ | 3.34 × 10⁻⁵-1.67 | 0.076 | 0.198 |  |  |
|  |  | Log(uniformity) | 6.88 × 10⁻¹² | 4.19 × 10⁻²⁷-1.13 × 10⁴ | 0.150 | 0.198 |  |  |
|  |  | Entropy | 1.61 × 10⁻⁷ | 2.31 × 10⁻¹⁷-1.13 × 10³ | 0.176 | 0.198 |  |  |
|  |  | TLG_mean_ | 1.04 | 0.984-1.11 | 0.160 | 0.198 |  |  |
|  | Fixed level threshold 50% above SUV_bkg_ | SUV_mean_ | 4.24 × 10⁻⁵ | 3.52 × 10⁻¹¹-51.0 | 0.159 | 0.351 | 65 | 2 |
|  |  | SUV_max_ | 62.7 | 6.87 × 10⁻²-5.73 × 10⁴ | 0.234 | 0.351 |  |  |
|  |  | MTV | 0.499 | 0.176-1.41 | 0.191 | 0.351 |  |  |
|  |  | SD | 2.03 × 10⁵ | 2.66 × 10⁻⁷-1.56 × 10¹⁷ | 0.381 | 0.490 |  |  |
|  |  | Skewness | 9.32 × 10⁻² | 1.12 × 10⁻⁶-7.77 × 10³ | 0.682 | 0.682 |  |  |
|  |  | Kurtosis | 7.94 | 3.39 × 10⁻²-1.86 × 10³ | 0.457 | 0.514 |  |  |
|  |  | Log(uniformity) | 1.75 × 10⁻²⁴ | 1.92 × 10⁻⁵⁶-1.60 × 10⁸ | 0.145 | 0.351 |  |  |
|  |  | Entropy | 8.59 × 10⁻¹⁶ | 2.34 × 10⁻³⁶-3.15 × 10⁵ | 0.151 | 0.351 |  |  |
|  |  | TLG_mean_ | 1.06 | 0.963-1.17 | 0.227 | 0.351 |  |  |
|  | Adaptive threshold with subtraction of SUV_bkg_ | SUV_mean_ | 0.109 | 2.13 × 10⁻⁴-55.8 | 0.486 | 0.746 | 64 | 3 |
|  |  | SUV_max_ | 4.65 | 4.16 × 10⁻²-520 | 0.523 | 0.746 |  |  |
|  |  | MTV | 0.776 | 0.507-1.19 | 0.243 | 0.746 |  |  |
|  |  | SD | 1.25 × 10⁻² | 5.96 × 10⁻⁹-2.60 × 10⁴ | 0.555 | 0.746 |  |  |
|  |  | Skewness | 2.30 × 10⁻² | 1.60 × 10⁻¹⁰-3.32 × 10⁶ | 0.694 | 0.746 |  |  |
|  |  | Kurtosis | 0.440 | 3.05 × 10⁻³-63.4 | 0.746 | 0.746 |  |  |
|  |  | Log(uniformity) | 4.13 × 10⁶ | 9.24 × 10⁻¹²-1.84 × 10²⁴ | 0.463 | 0.746 |  |  |
|  |  | Entropy | 834 | 3.32 × 10⁻⁸-2.10 × 10¹³ | 0.582 | 0.746 |  |  |
|  |  | TLG_mean_ | 1.04 | 0.976-1.10 | 0.249 | 0.746 |  |  |
| **Vue Point Sharp IR** | Fixed level threshold | SUV_mean_ | 17.9 | 1.88 × 10⁻²-1.69 × 10⁴ | 0.410 | 0.667 | 65 | 2 |
|  |  | SUV_max_ | 0.383 | 1.36 × 10⁻²-10.8 | 0.573 | 0.667 |  |  |
|  |  | MTV | 0.721 | 0.494-1.05 | 0.090 | 0.516 |  |  |
|  |  | SD | 4.89 × 10⁻³ | 1.92 × 10⁻⁹-1.25 × 10⁴ | 0.480 | 0.667 |  |  |
|  |  | Skewness | 80.4 | 8.41 × 10⁻⁶-7.68 × 10⁸ | 0.593 | 0.667 |  |  |
|  |  | Kurtosis | 1.72 | 4.08 × 10⁻²-72.5 | 0.777 | 0.777 |  |  |
|  |  | Log(uniformity) | 1.07 × 10⁸ | 3.52 × 10⁻¹⁶-3.24 × 10³¹ | 0.503 | 0.667 |  |  |
|  |  | Entropy | 4.86 × 10⁸ | 1.06 × 10⁻⁹-2.23 × 10²⁶ | 0.335 | 0.667 |  |  |
|  |  | TLG_mean_ | 1.04 | 0.991-1.09 | 0.115 | 0.516 |  |  |
|  | Adaptive threshold | SUV_mean_ | 0.189 | 1.14 × 10⁻²-3.13 | 0.244 | 0.371 | 62 | 5 |
|  |  | SUV_max_ | 3.97 | 0.311-50.6 | 0.289 | 0.371 |  |  |
|  |  | MTV | 0.679 | 0.400-1.15 | 0.151 | 0.371 |  |  |
|  |  | SD | 6.83 × 10⁻³ | 7.32 × 10⁻⁷-63.7 | 0.285 | 0.371 |  |  |
|  |  | Skewness | 222 | 1.12 × 10⁻²-4.38 × 10⁶ | 0.284 | 0.371 |  |  |
|  |  | Kurtosis | 5.22 × 10⁻³ | 1.34 × 10⁻⁵-2.04 | 0.084 | 0.371 |  |  |
|  |  | Log(uniformity) | 3.39 × 10⁻⁸ | 2.04 × 10⁻²⁴-5.63 × 10⁸ | 0.367 | 0.413 |  |  |
|  |  | Entropy | 5.90 × 10⁻⁵ | 2.08 × 10⁻¹⁵-1.68 × 10⁶ | 0.428 | 0.428 |  |  |
|  |  | TLG_mean_ | 1.04 | 0.981-1.10 | 0.192 | 0.371 |  |  |
|  | Fixed level threshold 50% above SUV_bkg_ | SUV_mean_ | 0.822 | 2.24 × 10⁻³-302 | 0.948 | 0.948 | 65 | 2 |
|  |  | SUV_max_ | 1.90 | 1.27 × 10⁻²-283 | 0.802 | 0.902 |  |  |
|  |  | MTV | 0.545 | 0.211-1.41 | 0.210 | 0.746 |  |  |
|  |  | SD | 3.26 × 10⁻³ | 1.07 × 10⁻¹⁰-9.94 × 10⁴ | 0.515 | 0.746 |  |  |
|  |  | Skewness | 36.6 | 2.67 × 10⁻⁴-5.03 × 10⁶ | 0.551 | 0.746 |  |  |
|  |  | Kurtosis | 2.99 × 10⁻² | 6.07 × 10⁻⁶-147 | 0.418 | 0.746 |  |  |
|  |  | Log(uniformity) | 4.61 × 10⁷ | 3.30 × 10⁻²⁰-6.44 × 10³⁴ | 0.580 | 0.746 |  |  |
|  |  | Entropy | 1.36 × 10⁵ | 4.37 × 10⁻¹³-4.23 × 10²² | 0.565 | 0.746 |  |  |
|  |  | TLG_mean_ | 1.06 | 0.962-1.16 | 0.246 | 0.746 |  |  |
|  | Adaptive threshold with subtraction of SUV_bkg_ | SUV_mean_ | 0.588 | 1.61 × 10⁻²-21.4 | 0.772 | 0.772 | 64 | 3 |
|  |  | SUV_max_ | 0.422 | 4.29 × 10⁻²-4.15 | 0.459 | 0.689 |  |  |
|  |  | MTV | 0.900 | 0.693-1.17 | 0.429 | 0.689 |  |  |
|  |  | SD | 39.9 | 1.04 × 10⁻²-1.53 × 10⁵ | 0.382 | 0.689 |  |  |
|  |  | Skewness | 360 | 9.81 × 10⁻⁴-1.32 × 10⁸ | 0.368 | 0.689 |  |  |
|  |  | Kurtosis | 0.201 | 5.79 × 10⁻³-6.99 | 0.376 | 0.689 |  |  |
|  |  | Log(uniformity) | 278 | 9.31 × 10⁻¹¹-8.29 × 10¹⁴ | 0.701 | 0.772 |  |  |
|  |  | Entropy | 23.3 | 1.06 × 10⁻⁷-5.15 × 10⁹ | 0.748 | 0.772 |  |  |
|  |  | TLG_mean_ | 1.02 | 0.981-1.06 | 0.309 | 0.689 |  |  |

CLM = number of colorectal liver metastases analysed, CLM missing = metastases with missing PET parameter for specific delineation algorithm

**Supplementary Table 6B. Pretreated colorectal liver metastases**

| **Reconstruction algorithm** | **Delineation algorithm** | **PET parameter** | **Odds** | | | | **CLM** | **CLM missing** |
| --- | --- | --- | --- | --- | --- | --- | --- | --- |
|  |  |  | **Odds ratio** | **95% CI** | **p-value** | **Adjusted p-value** |  |  |
| **Vue Point FX** | Fixed level threshold | SUV_mean_ | 7.17 × 10⁻⁴ | 6.08 × 10⁻⁹ – 8.45 × 10¹ | 0.224 | 0.763 | 64 | 5 |
|  |  | SUV_max_ | 1.65 × 10³ | 8.27 × 10⁻³ – 3.30 × 10⁸ | 0.234 | 0.763 |  |  |
|  |  | MTV | 1.57 × 10⁰ | 4.34 × 10⁻¹ – 5.71 × 10⁰ | 0.491 | 0.769 |  |  |
|  |  | SD | 6.02 × 10⁻¹⁰ | 3.13 × 10⁻²⁶ – 1.16 × 10⁷ | 0.267 | 0.763 |  |  |
|  |  | Skewness | 2.30 × 10⁻¹ | 2.97 × 10⁻⁵ – 1.78 × 10³ | 0.748 | 0.769 |  |  |
|  |  | Kurtosis | 2.34 × 10⁻¹ | 1.43 × 10⁻³ – 3.82 × 10¹ | 0.576 | 0.769 |  |  |
|  |  | Log(uniformity) | 5.14 × 10⁻⁶ | 3.07 × 10⁻²⁸ – 8.61 × 10¹⁶ | 0.641 | 0.769 |  |  |
|  |  | Entropy | 7.92 × 10⁻³ | 7.73 × 10⁻¹⁷ – 8.12 × 10¹¹ | 0.769 | 0.769 |  |  |
|  |  | TLG_mean_ | 7.96 × 10⁻¹ | 4.99 × 10⁻¹ – 1.27 × 10⁰ | 0.339 | 0.763 |  |  |
|  | Adaptive threshold | SUV_mean_ | 8.74 × 10⁻⁶ | 7.20 × 10⁻¹¹-1.06 | 0.051 | 0.248 | 62 | 6 |
|  |  | SUV_max_ | 8.28 × 10⁴ | 0.687-9.97 × 10⁹ | 0.058 | 0.248 |  |  |
|  |  | MTV | 1.88 | 0.420-8.42 | 0.409 | 0.526 |  |  |
|  |  | SD | 5.10 × 10⁻¹⁵ | 3.70 × 10⁻³¹-70.3 | 0.083 | 0.248 |  |  |
|  |  | Skewness | 0.366 | 1.53 × 10⁻⁴-876 | 0.800 | 0.800 |  |  |
|  |  | Kurtosis | 0.284 | 8.02 × 10⁻³-10.0 | 0.489 | 0.550 |  |  |
|  |  | Log(uniformity) | 5.46 × 10⁻¹⁰ | 1.37 × 10⁻²⁵-2.18 × 10⁶ | 0.245 | 0.370 |  |  |
|  |  | Entropy | 8.66 × 10⁻⁷ | 4.82 × 10⁻¹⁷-1.56 × 10⁴ | 0.247 | 0.370 |  |  |
|  |  | TLG_mean_ | 0.775 | 0.530-1.13 | 0.189 | 0.370 |  |  |
|  | Fixed level threshold 50% above SUV_bkg_ | SUV_mean_ | 6.59 × 10⁻² | 2.29 × 10⁻¹⁰-1.89 × 10⁷ | 0.784 | 0.963 | 62 | 5 |
|  |  | SUV_max_ | 24.4 | 6.85 × 10⁻⁵-8.69 × 10⁶ | 0.624 | 0.963 |  |  |
|  |  | MTV | 0.235 | 4.83 × 10⁻³-11.4 | 0.464 | 0.963 |  |  |
|  |  | SD | 6.63 × 10⁻⁷ | 4.80 × 10⁻²²-9.14 × 10⁸ | 0.424 | 0.963 |  |  |
|  |  | Skewness | 5.85 | 9.14 × 10⁻⁷-3.75 × 10⁷ | 0.825 | 0.963 |  |  |
|  |  | Kurtosis | 1.13 | 7.09 × 10⁻³-179 | 0.963 | 0.963 |  |  |
|  |  | Log(uniformity) | 35.9 | 1.54 × 10⁻²²-8.40 × 10²⁴ | 0.896 | 0.963 |  |  |
|  |  | Entropy | 690 | 3.15 × 10⁻¹⁴-1.51 × 10¹⁹ | 0.733 | 0.963 |  |  |
|  |  | TLG_mean_ | 1.02 | 0.552-1.90 | 0.939 | 0.963 |  |  |
|  | Adaptive threshold with subtraction of SUV_bkg_ | SUV_mean_ | 6.98 × 10⁻¹² | 6.59 × 10⁻²⁵-73.8 | 0.093 | 0.280 | 62 | 5 |
|  |  | SUV_max_ | 3.93 × 10⁹ | 1.65 × 10⁻²-9.34 × 10²⁰ | 0.098 | 0.280 |  |  |
|  |  | MTV | 8.11 | 0.192-343 | 0.273 | 0.351 |  |  |
|  |  | SD | 5.09 × 10⁻²⁴ | 3.60 × 10⁻⁵⁶-7.18 × 10⁸ | 0.156 | 0.280 |  |  |
|  |  | Skewness | 1.99 × 10⁻²⁵ | 1.23 × 10⁻⁴⁹-0.320 | **0.045*** | 0.280 |  |  |
|  |  | Kurtosis | 0.291 | 9.66 × 10⁻⁶-8.75 × 10³ | 0.814 | 0.814 |  |  |
|  |  | Log(uniformity) | 1.87 × 10²⁰ | 5.38 × 10⁻¹⁵-6.51 × 10⁵⁴ | 0.250 | 0.351 |  |  |
|  |  | Entropy | 7.62 × 10⁹ | 3.38 × 10⁻¹⁵-1.72 × 10³⁴ | 0.426 | 0.480 |  |  |
|  |  | TLG_mean_ | 0.360 | 9.38 × 10⁻²-1.38 | 0.136 | 0.280 |  |  |
| **Vue Point Sharp IR** | Fixed level threshold | SUV_mean_ | 7.29 × 10⁻⁶³ | 7.26 × 10⁻⁶³-7.31 × 10⁻⁶³ | **<0.001*** | **<0.001*** | 64 | 5 |
|  |  | SUV_max_ | 5.88 × 10⁶⁰ | 5.87 × 10⁶⁰-5.90 × 10⁶⁰ | **<0.001*** | **<0.001*** |  |  |
|  |  | MTV | 1.61 × 10⁶ | 1.60 × 10⁶-1.61 × 10⁶ | **<0.001*** | **<0.001*** |  |  |
|  |  | SD | 1.35 × 10⁻¹⁷⁶ | 1.35 × 10⁻¹⁷⁶-1.36 × 10⁻¹⁷⁶ | **<0.001*** | **<0.001*** |  |  |
|  |  | Skewness | 1.20 × 10³¹ | 1.20 × 10³¹-1.21 × 10³¹ | **<0.001*** | **<0.001*** |  |  |
|  |  | Kurtosis | 2.49 × 10⁻²⁸ | 2.48 × 10⁻²⁸-2.50 × 10⁻²⁸ | **<0.001*** | **<0.001*** |  |  |
|  |  | Log(uniformity) | 2.38 × 10⁻²⁶ | 2.38 × 10⁻²⁶-2.39 × 10⁻²⁶ | **<0.001*** | **<0.001*** |  |  |
|  |  | Entropy | 1.45 × 10⁻⁸ | 1.45 × 10⁻⁸-1.45 × 10⁻⁸ | **<0.001*** | **<0.001*** |  |  |
|  |  | TLG_mean_ | 3.74 × 10⁻³ | 3.73 × 10⁻³-3.76 × 10⁻³ | **<0.001*** | **<0.001*** |  |  |
|  | Adaptive threshold | SUV_mean_ | 1.59 × 10⁻³ | 7.06 × 10⁻⁷-3.59 | 0.102 | 0.260 | 62 | 6 |
|  |  | SUV_max_ | 1.01 × 10³ | 0.175-5.83 × 10⁶ | 0.118 | 0.260 |  |  |
|  |  | MTV | 3.15 | 0.534-18.6 | 0.205 | 0.308 |  |  |
|  |  | SD | 4.84 × 10⁻¹⁰ | 1.49 × 10⁻²²-1.57 × 10³ | 0.144 | 0.260 |  |  |
|  |  | Skewness | 1.09 | 1.63 × 10⁻²-72.9 | 0.968 | >0.999 |  |  |
|  |  | Kurtosis | 2.55 × 10⁻² | 3.12 × 10⁻⁴-2.09 | 0.103 | 0.260 |  |  |
|  |  | Log(uniformity) | 0.553 | 2.15 × 10⁻²²-1.42 × 10²¹ | 0.981 | >0.999 |  |  |
|  |  | Entropy | 0.982 | 8.15 × 10⁻¹⁵-1.18 × 10¹⁴ | >0.999 | >0.999 |  |  |
|  |  | TLG_mean_ | 0.687 | 0.417-1.13 | 0.141 | 0.260 |  |  |
|  | Fixed level threshold 50% above SUV_bkg_ | SUV_mean_ | 1.37 × 10⁻³ | 1.31 × 10⁻⁸-144 | 0.264 | 0.994 | 62 | 5 |
|  |  | SUV_max_ | 33.1 | 3.64 × 10⁻³-3.00 × 10⁵ | 0.452 | 0.994 |  |  |
|  |  | MTV | 0.510 | 1.75 × 10⁻³-148 | 0.816 | 0.994 |  |  |
|  |  | SD | 0.257 | 1.13 × 10⁻¹²-5.87 × 10¹⁰ | 0.919 | 0.994 |  |  |
|  |  | Skewness | 0.227 | 9.88 × 10⁻⁷-5.21 × 10⁴ | 0.814 | 0.994 |  |  |
|  |  | Kurtosis | 2.64 | 2.42 × 10⁻²-287 | 0.686 | 0.994 |  |  |
|  |  | Log(uniformity) | 2.38 × 10⁻⁴ | 4.79 × 10⁻³⁵-1.18 × 10²⁷ | 0.817 | 0.994 |  |  |
|  |  | Entropy | 0.853 | 1.74 × 10⁻¹⁹-4.18 × 10¹⁸ | 0.994 | 0.994 |  |  |
|  |  | TLG_mean_ | 0.890 | 0.282-2.81 | 0.842 | 0.994 |  |  |
|  | Adaptive threshold with subtraction of SUV_bkg_ | SUV_mean_ | 0.128 | 9.96 × 10⁻⁴-16.4 | 0.406 | 0.679 | 62 | 5 |
|  |  | SUV_max_ | 3.14 | 0.158-62.2 | 0.453 | 0.679 |  |  |
|  |  | MTV | 0.974 | 0.313-3.03 | 0.964 | 0.964 |  |  |
|  |  | SD | 0.135 | 9.51 × 10⁻⁷-1.91 × 10⁴ | 0.740 | 0.833 |  |  |
|  |  | Skewness | 2.93 × 10⁻⁷ | 6.46 × 10⁻¹⁵-13.2 | 0.094 | 0.679 |  |  |
|  |  | Kurtosis | 13.6 | 0.175-1.05 × 10³ | 0.240 | 0.679 |  |  |
|  |  | Log(uniformity) | 4.06 × 10⁷ | 1.03 × 10⁻⁷-1.59 × 10²² | 0.307 | 0.679 |  |  |
|  |  | Entropy | 3.39 × 10⁵ | 6.56 × 10⁻⁶-1.75 × 10¹⁶ | 0.312 | 0.679 |  |  |
|  |  | TLG_mean_ | 0.929 | 0.698-1.24 | 0.614 | 0.790 |  |  |

CLM = number of colorectal liver metastases analysed, CLM missing = metastases with missing PET parameter for specific delineation algorithm

**COX REGRESSION**

**Supplementary Table 7A + 7B. Univariate Cox regression in patients A) without oncological treatment prior imaging and liver surgery and B) with oncological treatment prior imaging and liver surgery, for associations with overall survival.**

| **Supplementary Table 7A. Chemonaïve patients** | | | | | | |  |  |  |
| --- | --- | --- | --- | --- | --- | --- | --- | --- | --- |
| **Reconstruction algorithm** | **Delineation algorithm** | **PET parameter** | **N** | **Event N** | **Mortality** | | | | |
|  |  |  |  |  | **HR** | **95% CI** | | **p-value** | **Adjusted p-value** |
| **Vue Point FX** | Fixed level threshold | SUV_mean_ | 40 | 18 | 0.82 | 0.63-1.07 | | 0.138 | 0.46 |
|  |  | SUV_max_ | 40 | 18 | 0.89 | 0.76-1.05 | | 0.169 | 0.46 |
|  |  | MTV | 40 | 18 | 1.01 | 0.99-1.02 | | 0.344 | 0.568 |
|  | Adaptive threshold | SUV_mean_ | 40 | 18 | 0.86 | 0.61-1.23 | | 0.416 | 0.624 |
|  |  | SUV_max_ | 40 | 18 | 0.89 | 0.76-1.05 | | 0.169 | 0.46 |
|  |  | MTV | 40 | 18 | 0.99 | 0.97-1.02 | | 0.553 | 0.699 |
|  | Fixed level threshold 50% above SUV_bkg_ | SUV_mean_ | 40 | 18 | 0.86 | 0.67-1.10 | | 0.23 | 0.46 |
|  |  | SUV_max_ | 40 | 18 | 0.90 | 0.77-1.06 | | 0.198 | 0.46 |
|  |  | MTV | 40 | 18 | 1.00 | 0.98-1.03 | | 0.989 | 0.989 |
|  | Adaptive threshold with subtraction of SUV_bkg_ | SUV_mean_ | 40 | 18 | 0.94 | 0.67-1.30 | | 0.69 | 0.828 |
|  |  | SUV_max_ | 40 | 18 | 0.90 | 0.77-1.05 | | 0.19 | 0.46 |
|  |  | MTV | 40 | 18 | 1.00 | 0.98-1.01 | | 0.989 | 0.989 |
| **Vue Point Sharp IR** | Fixed level threshold | SUV_mean_ | 40 | 18 | 0.85 | 0.68-1.07 | | 0.165 | 0.46 |
|  |  | SUV_max_ | 40 | 18 | 0.91 | 0.80-1.03 | | 0.145 | 0.46 |
|  |  | MTV | 40 | 18 | 1.01 | 0.99-1.02 | | 0.355 | 0.568 |
|  | Adaptive threshold | SUV_mean_ | 40 | 18 | 0.82 | 0.58-1.16 | | 0.27 | 0.498 |
|  |  | SUV_max_ | 40 | 18 | 0.91 | 0.80-1.03 | | 0.123 | 0.46 |
|  |  | MTV | 40 | 18 | 1.00 | 0.98-1.02 | | 0.738 | 0.843 |
|  | Fixed level treshold 50% above SUV_bkg_ | SUV_mean_ | 40 | 18 | 0.87 | 0.70-1.09 | | 0.224 | 0.46 |
|  |  | SUV_max_ | 40 | 18 | 0.91 | 0.80-1.03 | | 0.123 | 0.46 |
|  |  | MTV | 40 | 18 | 1.00 | 0.98-1.03 | | 0.971 | 0.989 |
|  | Adaptive treshold with subtraction of SUV_bkg_ | SUV_mean_ | 40 | 18 | 0.89 | 0.64-1.25 | | 0.513 | 0.684 |
|  |  | SUV_max_ | 40 | 18 | 0.91 | 0.80-1.03 | | 0.123 | 0.46 |
|  |  | MTV | 40 | 18 | 1.00 | 0.98-1.01 | | 0.46 | 0.649 |

N = number of patients, Event N = number of subjects who had the event (death), HR = hazard ratio

| **Supplementary Table 7B. Pretreated patients** | | | | | | |  |  |  |
| --- | --- | --- | --- | --- | --- | --- | --- | --- | --- |
| **Reconstruction algorithm** | **Delineation algorithm** | **PET parameter** | **N** | **Event N** | **Mortality** | | | | |
|  |  |  |  |  | **HR** | **95% CI** | | **p-value** | **Adjusted p-value** |
| **Vue Point FX** | Fixed level threshold | SUV_mean_ | 28 | 22 | 1.11 | 0.92-1.33 | | 0.28 | 0.539 |
|  |  | SUV_max_ | 28 | 22 | 1.05 | 0.93-1.18 | | 0.429 | 0.539 |
|  |  | MTV | 28 | 22 | 1.00 | 0.99-1.01 | | 0.46 | 0.539 |
|  | Adaptive threshold | SUV_mean_ | 27 | 22 | 1.18 | 0.88-1.59 | | 0.261 | 0.539 |
|  |  | SUV_max_ | 27 | 22 | 1.03 | 0.91-1.17 | | 0.632 | 0.632 |
|  |  | MTV | 27 | 22 | 1.01 | 0.98-1.05 | | 0.44 | 0.539 |
|  | Fixed level threshold 50% above SUV_bkg_ | SUV_mean_ | 28 | 22 | 1.07 | 0.88-1.30 | | 0.505 | 0.543 |
|  |  | SUV_max_ | 28 | 22 | 1.05 | 0.94-1.18 | | 0.392 | 0.539 |
|  |  | MTV | 28 | 22 | 1.03 | 0.99-1.08 | | 0.133 | 0.539 |
|  | Adaptive threshold with subtraction of SUV_bkg_ | SUV_mean_ | 28 | 22 | 1.19 | 0.85-1.67 | | 0.31 | 0.539 |
|  |  | SUV_max_ | 28 | 22 | 1.05 | 0.94-1.19 | | 0.379 | 0.539 |
|  |  | MTV | 28 | 22 | 1.01 | 0.99-1.02 | | 0.284 | 0.539 |
| **Vue Point Sharp IR** | Fixed level threshold | SUV_mean_ | 28 | 22 | 1.10 | 0.94-1.29 | | 0.233 | 0.539 |
|  |  | SUV_max_ | 28 | 22 | 1.05 | 0.96-1.14 | | 0.329 | 0.539 |
|  |  | MTV | 28 | 22 | 1.00 | 0.99-1.01 | | 0.46 | 0.539 |
|  | Adaptive threshold | SUV_mean_ | 27 | 22 | 1.18 | 0.91-1.52 | | 0.209 | 0.539 |
|  |  | SUV_max_ | 27 | 22 | 1.03 | 0.94-1.13 | | 0.520 | 0.543 |
|  |  | MTV | 27 | 22 | 1.01 | 0.98-1.04 | | 0.456 | 0.539 |
|  | Fixed level treshold 50% above SUV_bkg_ | SUV_mean_ | 28 | 22 | 1.07 | 0.90-1.27 | | 0.472 | 0.539 |
|  |  | SUV_max_ | 28 | 22 | 1.05 | 0.96-1.14 | | 0.329 | 0.539 |
|  |  | MTV | 28 | 22 | 1.03 | 0.99-1.08 | | 0.133 | 0.539 |
|  | Adaptive treshold with subtraction of SUV_bkg_ | SUV_mean_ | 28 | 22 | 1.17 | 0.85-1.61 | | 0.338 | 0.539 |
|  |  | SUV_max_ | 28 | 22 | 1.05 | 0.96-1.14 | | 0.329 | 0.539 |
|  |  | MTV | 28 | 22 | 1.01 | 1.0-1.02 | | 0.262 | 0.539 |

N = number of subjects, Event N = number of subjects who had the event (death), HR = hazard ratio

**Supplementary Table 8A + 8B. Multivariate Cox regression patients A) without oncological treatment prior imaging and liver surgery and B) with oncological treatment prior imaging and liver surgery, for associations with overall survival.**

| **Supplementary Table 8A. Chemonaïve patients** | | | | | | | |  |
| --- | --- | --- | --- | --- | --- | --- | --- | --- |
| **Reconstruction algorithm** | **Delineation algorithm** | **PET parameter** | **N** | **Event N** | **Mortality** | | | |
|  |  |  |  |  | **HR** | **95% CI** | **p-value** | **Adjusted p-value** |
| **Vue Point FX** | Fixed level threshold | SUV_mean_ | 40 | 18 | 0.24 | 0.03-2.20 | 0.207 | 0.382 |
|  |  | SUV_max_ | 40 | 18 | 2.10 | 0.53-8.32 | 0.290 | 0.462 |
|  |  | MTV_tot_ | 40 | 18 | 1.01 | 1.00-1.03 | 0.149 | 0.325 |
|  | Adaptive threshold | SUV_mean_ | 40 | 18 | 2.48 | 0.83-7.43 | 0.104 | 0.277 |
|  |  | SUV_max_ | 40 | 18 | 0.61 | 0.36-1.02 | 0.061 | 0.219 |
|  |  | MTV_tot_ | 40 | 18 | 1.00 | 0.97-1.03 | 0.964 | 0.964 |
|  | Fixed level threshold 50% above SUV_bkg_ | SUV_mean_ | 40 | 18 | 3.32 | 0.33-33.4 | 0.308 | 0.462 |
|  |  | SUV_max_ | 40 | 18 | 0.41 | 0.09-1.81 | 0.239 | 0.41 |
|  |  | MTV_tot_ | 40 | 18 | 1.01 | 0.99-1.04 | 0.330 | 0.466 |
|  | Adaptive threshold with subtraction of SUV_bkg_ | SUV_mean_ | 40 | 18 | 1.95 | 0.96-3.97 | 0.064 | 0.219 |
|  |  | **SUV_max_** | **40** | **18** | **0.65** | **0.43-0.99** | **0.047*** | 0.219 |
|  |  | MTV_tot_ | 40 | 18 | 1.00 | 0.99-1.02 | 0.827 | 0.902 |
| **Vue Point Sharp IR** | Fixed level threshold | SUV_mean_ | 40 | 18 | 1.22 | 0.44-3.35 | 0.698 | 0.798 |
|  |  | SUV_max_ | 40 | 18 | 0.80 | 0.45-1.42 | 0.445 | 0.548 |
|  |  | MTV_tot_ | 40 | 18 | 1.01 | 1.0-1.03 | 0.179 | 0.358 |
|  | Adaptive threshold | SUV_mean_ | 40 | 18 | 1.42 | 0.65-3.10 | 0.378 | 0.504 |
|  |  | SUV_max_ | 40 | 18 | 0.78 | 0.57-1.07 | 0.127 | 0.305 |
|  |  | MTV_tot_ | 40 | 18 | 1.01 | 0.99-1.03 | 0.457 | 0.548 |
|  | Fixed level threshold 50% above SUV_bkg_ | SUV_mean_ | 40 | 18 | 3.04 | 0.94-9.77 | 0.063 | 0.219 |
|  |  | **SUV_max_** | **40** | **18** | **0.46** | **0.23-0.95** | **0.035*** | 0.219 |
|  |  | MTV_tot_ | 40 | 18 | 1.03 | 1.00-1.06 | 0.093 | 0.277 |
|  | Adaptive threshold with subtraction of SUV_bkg_ | **SUV_mean_** | **40** | **18** | **2.69** | **1.01-7.18** | **0.048*** | 0.219 |
|  |  | **SUV_max_** | **40** | **18** | **0.63** | **0.42-0.95** | **0.026*** | 0.219 |
|  |  | MTV_tot_ | 40 | 18 | 1.00 | 0.98-1.02 | 0.887 | 0.926 |

N = number of subjects, Event N = number of subjects who had the event (death), HR = hazard ratio

| **Supplementary Table 8B. Pretreated patients** | | | | | | | | |
| --- | --- | --- | --- | --- | --- | --- | --- | --- |
| **Reconstruction algorithm** | **Delineation algorithm** | **PET parameter** | **N** | **Event N** | **Mortality** | | | |
|  |  |  |  |  | **HR** | **95% CI** | **p-value** | **Adjusted p-value** |
| **Vue Point FX** | Fixed level threshold | SUV_mean_ | 28 | 22 | 1.51 | 0.80-2.87 | 0.202 | 0.606 |
|  |  | SUV_max_ | 28 | 22 | 0.80 | 0.52-1.24 | 0.314 | 0.685 |
|  |  | MTV_tot_ | 28 | 22 | 1.00 | 0.99-1.02 | 0.494 | 0.788 |
|  | Adaptive threshold | SUV_mean_ | 27 | 22 | 1.76 | 0.89-3.51 | 0.107 | 0.571 |
|  |  | SUV_max_ | 27 | 22 | 0.78 | 0.52-1.17 | 0.235 | 0.627 |
|  |  | MTV_tot_ | 27 | 22 | 1.02 | 0.96-1.09 | 0.525 | 0.788 |
|  | Fixed level threshold 50% above SUV_bkg_ | SUV_mean_ | 28 | 22 | 0.18 | 0.03-1.14 | 0.068 | 0.571 |
|  |  | SUV_max_ | 28 | 22 | 2.81 | 0.92-8.60 | 0.071 | 0.571 |
|  |  | MTV_tot_ | 28 | 22 | 1.03 | 0.97-1.09 | 0.308 | 0.685 |
|  | Adaptive threshold with subtraction of SUV_bkg_ | SUV_mean_ | 28 | 22 | 1.16 | 0.43-3.09 | 0.77 | 0.841 |
|  |  | SUV_max_ | 28 | 22 | 0.97 | 0.70-1.35 | 0.873 | 0.885 |
|  |  | MTV_tot_ | 28 | 22 | 1.00 | 0.98- 1.03 | 0.669 | 0.839 |
| **Vue Point Sharp IR** | Fixed level threshold | SUV_mean_ | 28 | 22 | 1.19 | 0.75-1.87 | 0.465 | 0.788 |
|  |  | SUV_max_ | 28 | 22 | 0.95 | 0.73-1.24 | 0.699 | 0.839 |
|  |  | MTV_tot_ | 28 | 22 | 1.00 | 0.99-1.01 | 0.663 | 0.839 |
|  | Adaptive threshold | SUV_mean_ | 27 | 22 | 1.37 | 0.87-2.18 | 0.176 | 0.606 |
|  |  | SUV_max_ | 27 | 22 | 0.92 | 0.75-1.12 | 0.402 | 0.788 |
|  |  | MTV_tot_ | 27 | 22 | 1.01 | 0.98-1.04 | 0.433 | 0.788 |
|  | Fixed level threshold 50% above SUV_bkg_ | SUV_mean_ | 28 | 22 | 0.43 | 0.15-1.24 | 0.119 | 0.571 |
|  |  | SUV_max_ | 28 | 22 | 1.55 | 0.90-2.66 | 0.116 | 0.571 |
|  |  | MTV_tot_ | 28 | 22 | 1.03 | 0.98-1.09 | 0.184 | 0.606 |
|  | Adaptive threshold with subtraction of SUV_bkg_ | SUV_mean_ | 28 | 22 | 0.94 | 0.42-2.11 | 0.885 | 0.885 |
|  |  | SUV_max_ | 28 | 22 | 1.03 | 0.85-1.24 | 0.771 | 0.841 |
|  |  | MTV_tot_ | 28 | 22 | 1.01 | 0.98-1.03 | 0.567 | 0.8 |

N = number of subjects, Event N = number of subjects who had the event (death), HR = hazard ratio

**Supplementary Fig. 2** Kaplan-Meier curves presenting overall survival for patients included divided by predominant histopathological growth pattern (HGP) in A) chemonaïve patients and B) pretreated patients.
eHGP = encapsulating HGP and rHGP = replacement HGP.
